# Cultivable Bacterial Microbiota of Northern Bobwhite (*Colinus virginianus*): A New Reservoir of Antimicrobial Resistance?

**DOI:** 10.1371/journal.pone.0099826

**Published:** 2014-06-17

**Authors:** Hongwen Su, Jessica McKelvey, Dale Rollins, Michael Zhang, Donald J. Brightsmith, James Derr, Shuping Zhang

**Affiliations:** 1 Department of Veterinary Pathobiology, College of Veterinary Medicine and Biomedical Sciences, Texas A&M University, College Station, Texas, United States of America; 2 Rolling Plains Quail Research Foundation, Texas AgriLife Research, San Angelo, Texas, United States of America; Institut National de la Recherche Agronomique, France

## Abstract

The northern bobwhite (*Colinus virginianus*) is an ecologically and economically important avian species. At the present time, little is known about the microbial communities associated with these birds. As the first step to create a quail microbiology knowledge base, the current study conducted an inventory of cultivable quail tracheal, crop, cecal, and cloacal microbiota and associated antimicrobial resistance using a combined bacteriology and DNA sequencing approach. A total of 414 morphologically unique bacterial colonies were selected from nonselective aerobic and anaerobic cultures, as well as selective and enrichment cultures. Analysis of the first 500-bp 16S rRNA gene sequences in conjunction with biochemical identifications revealed 190 non-redundant species-level taxonomic units, representing 160 known bacterial species and 30 novel species. The bacterial species were classified into 4 phyla, 14 orders, 37 families, and 59 or more genera. *Firmicutes* was the most commonly encountered phylum (57%) followed by *Actinobacteria* (24%), *Proteobacteria* (17%) and *Bacteroidetes* (0.02%). Extensive diversity in the species composition of quail microbiota was observed among individual birds and anatomical locations. Quail microbiota harbored several opportunistic pathogens, such as *E. coli* and *Ps. aeruginosa*, as well as human commensal organisms, including *Neisseria* species. Phenotypic characterization of selected bacterial species demonstrated a high prevalence of resistance to the following classes of antimicrobials: phenicol, macrolide, lincosamide, quinolone, and sulphate. Data from the current investigation warrant further investigation on the source, transmission, pathology, and control of antimicrobial resistance in wild quail populations.

## Introduction

The northern bobwhite (*Colinus virginianus*) is an ecologically and economically important natural resource in the United States. A survey conducted by the U.S. Fish and Wildlife Service in 2011 indicates that bobwhite is one of the most preferred small game species pursued by over 840,000 hunters on over 9 million hunting days in 2011 [1]. Over the past several decades, bobwhite populations have been declining across their range. In the state of Texas alone, bobwhites have been diminishing at an average annual rate of 5.6% since 1980 which has resulted in a 75% population decline [2]. Although habitat loss associated with intensive agricultural practices has been considered as a major contributor, quail declines have continued even in areas of apparently suitable habitat during favorable weather conditions [3]. The involvement of infectious agents has been investigated, but studies have failed to identify any pathogens with the potential to kill large numbers of quail [4], [5]. This ongoing decline, termed “Idiopathic Quail Decline” underscores the need for a holistic approach to the study of quail health [3], [6].

One important aspect of animal health that is just beginning to be understood among many vertebrate species is the indigenous microbial populations or the microbiota. Recent investigations have shown that the host’s nutritional, physiological, and immunological processes are profoundly affected by the microbiota [7], [8], [9]. However, the composition of the microbiota is also influenced by factors such as the host’s genetics, diet, environment, and infection status [7], [9]. The gut microbiota contributes to the digestion and fermentation of carbohydrates, the development of gut-associated lymphoid tissues (GALTs), and the prevention of colonization by pathogens [10].

Over the past two decades, numerous culture-dependent and culture-independent microbiome studies have been carried out to characterize the composition and dynamic changes of intestinal microbiota of mammals, domestic poultry, and several wild avian species [11], [12], [13], [14], [15]. However, the microbiota of northern bobwhite remains largely unexplored. Documenting the microbiota of this species is important because it may identify agents that could contribute to the ongoing population declines. In addition, it is important to document the presence and extent of antimicrobial resistance in bobwhite bacterial microbiota because wild birds could carry drug resistant bacteria potentially pathogenic to humans and other animal species [16], [17]. Although wild bobwhite populations do not have contact with antibiotics as therapeutic agents, exposure via water and food contaminated by antimicrobial resistant bacteria originating from human waste or farm-reared quail is likely [18], [19], [20]. Previously, multidrug resistant *Salmonella enterica* serovar Typhimurium was isolated from diseased bobwhites [21]. Resistance to tetracycline was detected in *Edwardsiella hoshinae* isolated from healthy northern bobwhite quail [22]. However, no study has been conducted to determine the susceptibility or resistance patterns of other bacterial species commonly found in avian and mammalian gastrointestinal tracts. If bobwhites do carry drug-resistant organisms as part of their microbiota, they may serve as a reservoir for antimicrobial resistant bacteria or resistance genes that can be transmitted to other wild animals or to humans upon handling and consumption.

To fill the gaps in existing knowledge relating to bobwhite microbiology, the current investigation characterized cultivable tracheal and gastrointestinal microbiota of wild northern bobwhite and determined the antimicrobial susceptibility and resistance patterns of selected commensal and opportunistic pathogenic bacterial species.

## Materials and Methods

### Sample Collection and Processing

Sample collection and processing were completed by the Central Specimen Receiving, Processing and Distribution Laboratory (CSRPDL) of the Institute of Environmental and Human Health, Texas Tech University. Animal and tissue uses were approved by the Institutional Animal Care and Use Committees of Texas A&M University (IACUC 2011-193) and Texas Tech University (IACUC 11049-07). A total of 49 live-trapped bobwhites throughout the Rolling Plains Ecoregion were used in this study. Due to sample sharing among multiple investigators, only 49 trachea, 48 crops, 39 ceca, and 38 cloacal swabs were available for microbiological evaluations. All trapping practices were conducted under the auspices of a Texas Parks & Wildlife Department Scientific Collector’s permit. Each bird was labeled with a unique 6-digit ID number while the sex and age were determined by trained personnel using plumage characteristics. The age of three birds was not labeled due to unknown reasons. Tissue and swab samples in Cary-Blair transport medium (BD, Franklin Lakes, NJ, USA) were transported from the field on ice to CSRPDL within 8 hours. All samples were stored at −20°C until testing (within 2 months). Prior to culture, trachea, crops, and ceca were thawed and cut open aseptically.

### Cultivation of Bacteria

The luminal contents and/or mucosal surfaces of trachea, crops, and ceca were sampled with sterile polyester swabs (Puritan Medical, Guilford, Maine, USA). Tracheal samples were inoculated onto the surface of solid media including Tryptic Soy Agar with 5% Sheep Blood (TSA), Columbia Colistin Nalidixic Acid agar with 5% Sheep Blood (CNA), Anaerobic Phenylethyl Alcohol Blood agar (PEA), MacConkey agar (MAC), and Lowenstein-Jensen Medium Slants (LJ); and into Brain Heart Infusion broth (BHI) and Mycoplasma broth (Frey). Crop, cecal, and cloacal samples were inoculated on to TSA, CNA, PEA, MAC, and Xylose-lysine-tergitol 4 (XLT4) as well in Brain Heart Infusion broth (BHI) and Buffered Peptone Water (BPW). Aerobic cultures (TSA, CNA, LJ, BHI, and Frey) were incubated at 37°C in either 5% CO_2_ or ambient air (MAC, BPW, and XLT4). Anaerobic cultures (TSA, PEA, and BHI) were incubated at 37°C in anaerobic jars with disposable gas packs (Remel Inc., Lenexa, Kansas, USA). Cultures (TSA, CNA, MAC, PEA, XLT4) were examined daily for growth up to 5 days. Mycoplasma cultures (Frey) and mycobacteria cultures (LJ) were incubated for 14 days. BHI and BPW cultures were subcultured after overnight incubation onto TSA and XLT4, respectively. Morphologically unique bacterial colonies were subcultured under appropriate conditions to obtain pure cultures.

### Bacterial Identification and Antimicrobial Susceptibility Testing

Partial **c**haracterization of bacterial strains was also performed by biochemical tests, including Gram staining; production of oxidase, catalase, urease; motility, and indole test. Selected biochemical reactions from several bacterial ID kits, including RapID CB Plus, RapID ANA II, RapID NF Plus, RapID One, RapID Staph, and RapID STR, were also used to confirm identity. Pure bacterial cultures were subjected to 16S rRNA gene sequencing. To prepare DNA template for PCR, isolated colonies (1 to 2) were resuspended in 100 µl of ddH2O and boiled for 10 min. The bacterial broad-range 16S rDNA primer pairs: fD1mod (5′-AGAGTTTGATCYTGGYYYAG-3′, corresponding to positions 8–27 in the *E. coli* 16S rRNA gene) and 16S1RR-B (5′-CTTTACGCCCARTRAWTCCG-3′, corresponding to positions of 556–575) [23] were used to amplify the 5′ end of the 16S rRNA gene. The thermal cycling conditions were as follows: an initial denaturation at 94°C for 3 min; 35 cycles of 94°C for 30 sec, 56°C for 30 sec, and 72°C for 30 sec; and a final extension step at 72°C for 7 min. A portion of each PCR product was subjected to electrophoresis on 1.5% agarose gel and visualized using the AlphaImager Gel Documentation system (ProteinSimple, Santa Clara, CA). The PCR products were purified and sequenced using the 16S1RR-B primer. The resultant sequences were subjected to homology search against GenBank database by using the BLAST search algorithm as well as MicroSeq ID 2.0 500-bp library (Applied Biosystems, Foster, CA). Sequence identities of ≥99% and ≥97% were used as criteria for species and genus assignments, respectively. The sequences sharing less than 97% identities with the 16S rRNA sequences in the databases were considered as potentially new species [24].

Selected bacterial species were subjected to antimicrobial susceptibility testing using the Sensititer Veterinary Susceptibility Plates, AVIAN1F, (Trek Diagnostic Systems, Westlake, OH). The inoculated antimicrobial plates were incubated at 37°C in ambient air for 24 h. The antimicrobial agents tested included amoxicillin, ceftiofur, clindamycin, enrofloxacin, erythromycin, florfenicol, gentamicin, neomycin, novobiocin, oxytetracycline, penicillin, spectinomycin, streptomycin, sulphadimethoxine, sulphatiazole, tetracycline, trimethoprim/sulpha, and tylosin. The results were interpreted by using Clinical Laboratory Standards Institute (CLSI) guidelines for broth microdilution methods [25]. *E. coli* ATTC 25922 and *Ps. aeruginosa* ATCC 27853 were used as the quality control strains.

### Statistical Analysis

To determine if the number of bacterial species found in each sample varied with regard to sex, age, tissue type or sex by age interaction we used a general linear model with a Poisson distribution. To determine if the distribution of bacterial species varied with tissue type, sex, age and the sex by age interaction term we used a binomial logistic regression for all bacterial species which were detected in 15 or more different tissues. Binomial logistic regression was used to determine if the distribution of bacterial genera varied by tissue type, sex, age and the sex by age interaction for all genera which were detected in 15 or more tissue samples analyzed. These analyses included data from 45 healthy bobwhites for which age class and sex were known. Data from one Scaled Quail (*Callipepla squamata*) and one sick Northern Bobwhite were omitted along with data from two birds for which age class was unknown and one for which age, sex and species were unknown. For all analyses we used an α = 0.05.

### Nucleotide Sequence Accession Numbers

The sequences of individual bacterial species were deposited in the Genbank database under accession numbers (KJ023256 to KJ023442).

## Results

### The Composition of Cultivable Bacterial Microbiota

A total of 828 representative bacterial colonies were collected from nonselective aerobic and anaerobic cultures, as well as selective and enrichment cultures for *Mycoplasma* spp., *Mycobacterium* spp., and *Salmonella enterica* serovars. Bacterial colonies were screened based on their morphological, growth, and biochemical characteristics. When two or more colonies showed identical morphology and biochemical characteristics, only one of these colonies was sequenced. In total, 414 distinct colonies were subjected to 16S rRNA gene sequencing which revealed 190 nonredundant species-level taxonomic units. One hundred-sixty (84%) sequences shared 99% to 100% similarities to the 16S rRNA gene sequences deposited in GenBank, therefore representing known bacterial species. In addition, 9 (4.74%) sequences showed 97% to 98% similarities and 21 (11%) sequences had less than 97% of similarities to previously reported 16S rRNA gene sequences, suggesting that these were novel species and genera (Table S1). The sequences of all species level taxa have been submitted to Genbank.

The quail bacterial species could be classified into 4 phyla, 14 orders, 38 families, 61 genera, and 160 known species and potentially 30 novel species (Table 1). *Firmicutes* (56%) was the largest phylum, followed by *Actinobacteria* (24%), *Proteobacteria* (18%) and *Bacteroidetes* (0.02%). At family level, the vast majority of bacterial species belonged to *Bacillaceae* (22%), *Paenibacillaceae* (11%), *Enterobacteriaceae* (11%), *Microbacteriaceae* (6.84%), *Lactobacillaceae* (5.79%), and *Streptococcaceae* (5.79%).

**Table 1:** The composition of quail cultivable bacterial microbiota*.

Phylum	Order	Family	Genus #	Species #
*Firmicutes* (56.32%)	*Bacillales* (34.21%)	*Bacillaceae* (21.58%)	5	41
		*Paenibacillaceae* (10.53%)	2	20
		*Staphylococcaceae* (2.11%)	1	4
	*Lactobacillales* (16.32%)	*Lactobacillaceae* (5.79%)	2	11
		*Streptococcaceae* (5.79%)	3	11
		*Enterococcaceae* (4.21%)	1	8
		*Leuconostocaceae* (0.53%)	1	1
	*Clostridiales* (5.26%)	*Clostridiaceae* (1.58%)	1	3
		*Eubacteriaceae* (1.58%)	1	3
		*Lachnospiraceae* (1.05%)	1	2
		*Clostridiales* Family XI (1.05%)	2	2
	*Selenomonadales* (0.53%)	*Veillonellaceae* (0.53%)	1	1
*Actinobacteria* (24.21%)	*Actinomycetales* (23.68%)	*Microbacteriaceae* (6.84%)	4	13
		*Streptomycetaceae* (5.26%)	1	10
		*Micrococcaceae* (6.84%)	3	7
		*Corynebacteriaceae* (1.58%)	1	3
		*Nocardiaceae* (1.58%)	1	3
		*Actinomycetaceae* (1.05%)	2	2
		*Promicromonosporaceae* (1.05%)	1	2
		*Gordoniaceae* (0.53%)	1	1
		*Micromonosporaceae* (0.53%)	1	1
		*Propionibacteriaceae* (0.53%)	1	1
		*Mycobacteriaceae* (0.53%)	1	1
		*Pseudonocardiaceae* (0.53%)	1	1
	*Bifidobacteriales* (0.53%)	*Bifidobacteriaceae* (0.53%)	1	1
*Proteobacteria* (17.89%)	*Enterobacteriales* (10.53%)	*Enterobacteriaceae* (10.53%)	9	20
	*Pseudomonadales* (2.63%)	*Moraxellaceae* (2.11%)	1	4
		*Pseudomonadaceae* (0.53%)	1	1
	*Burkholderiales* (1.58%)	*Alcaligenaceae* (0.53%)	1	1
		*Burkholderiaceae* (0.533%)	1	1
		*Ralstoniaceae* (0.53%)	1	1
	*Neisseriales* (1.58%)	*Neisseriaceae* (1.58%)	1	3
	*Rhizobiales* (1.05%)	*Brucellaceae* (0.53%)	1	1
		*Rhizobiaceae* (0.53%)	1	1
	*Xanthomonadales* (0.53%)	*Xanthomonadaceae* (0.53%)	1	1
*Bacteroidetes* (0.02%)	*Bacteroidales* (1.05%)	*Bacteroidaceae* (0.53%)	1	1
		*Porphyromonadaceae* (0.53%)	1	1
	*Flavobacteriales* (0.53%)	*Flavobacteriaceae* (0.53%)	1	1
Total	14	38	61	190

*Shown are the numbers (percentages) of order, family, genus, and species. A total of 49 bobwhites were used in this study. Bacteria were isolated from trachea, crops, ceca, and cloaca. Species or genus-level identifications were achieves using 16S rRNA gene sequencing and biochemistry. Highest Blast hits were from cultured bacteria.

### Composition of Tissue-specific Cultivable Bacterial Microbiota

The tracheal microbiota consisted of 21 species belonging to 12 families with *Lactobacillaceae* (19%) being the most commonly encountered bacterial family. The crop microbiota consisted of 90 species in 20 families with *Bacillaceae* (21%) and *Enterobacteriaceae* (18%) being the top 2 families. The cecal microbiota consisted of 69 species from 22 families with *Bacillaceae* (29%), *Enterococcaceae* (10%), and *Paenibacillaceae* (10%) being the top 3 families. The cloacal microbiota was composed of 71 species belonging to 19 families with *Bacillaceae* (28%) and *Streptococcaceae* (13%) being the majority (Table 2).

**Table 2:** Tissue-specific composition of quail microbiota*.

Phylum	Family	Number of Species			
		Trachea	Crop	Ceca	Cloaca
*Firmicutes* (56.32%)	*Bacillaceae*	2 (9.52%)	19 (21.11%)	20 (28.99%)	20 (28.17%)
	*Clostridiaceae*			3 (4.35%)	2 (2.82%)
	*Enterococcaceae*	1 (4.76%)	2 (2.22%)	7 (10.14%)	5 (7.04%)
	*Eubacteriaceae*			3 (4.35%)	
	*Lachnospiraceae*			2 (2.90%)	
	*Lactobacillaceae*	4 (19.05%)	8 (8.89%)	3 (4.35%)	1 (1.41%)
	*Leuconostocaceae*		1 (1.11%)		
	*Paenibacillaceae*	2 (9.52%)	10 (11.11%)	7 (10.14%)	4 (5.63%)
	*Clostridiales* Family XI				2 (2.82%)
	*Staphylococcaceae*		3 (3.33%)	1 (1.45%)	3 (4.23%)
	*Streptococcaceae*		2 (2.22%)	4 (5.80%)	9 (12.68%)
	*Veillonellaceae*				1 (1.41%)
*Actinobacteria* (24.21%)	*Actinomycetaceae*			1 (1.45%)	1 (1.41%)
	*Bifidobacteriaceae*			1 (1.45%)	1 (1.41%)
	*Corynebacteriaceae*		1 (1.11%)		2 (2.82%)
	*Gordoniaceae*		1 (1.11%)		
	*Microbacteriaceae*	3 (14.29%)	6 (6.67%)	1 (1.45%)	6 (8.45%)
	*Micrococcaceae*		2 (2.22%)	2 (2.90%)	5 (7.04%)
	*Micromonosporaceae*		1 (1.11%)		
	*Mycobacteriaceae*	1 (4.76%)			
	*Nocardiaceae*		3 (3.33%)		
	*Promicromonosporaceae*		2 (2.22%)		
	*Propionibacteriaceae*				1 (1.41%)
	*Pseudonocardiaceae*				1 (1.41%)
	*Streptomycetaceae*	2 (9.52%)	6 (6.67%)	1 (1.45%)	2 (2.82%)
*Proteobacteria* (17.89%)	*Alcaligenaceae*		1 (1.11%)		
	*Brucellaceae*				1 (1.41%)
	*Burkholderiaceae*	1 (4.76%)			
	*Enterobacteriaceae*	2 (9.52%)	16 (17.78%)	5 (7.25%)	1 (1.41%)
	*Moraxellaceae*	1 (4.76%)	4 (4.44%)	2 (2.90%)	
	*Neisseriaceae*			1 (1.45%)	3 (4.23%)
	*Pseudomonadaceae*	1 (4.76%)	1 (1.11%)	1 (1.45%)	
	*Ralstoniaceae*	1 (4.76%)			
	*Rhizobiaceae*			1 (1.45%)	
	*Xanthomonadaceae*			1 (1.45%)	
*Bacteroidetes* (0.02%)	*Bacteroidaceae*			1 (1.45%)	
	*Flavobacteriaceae*		1 (1.11%)		
	*Porphyromonadaceae*			1 (1.45%)	
Total		21 (100%)	90 (100%)	69 (100%)	71 (100%)

*Shown are the numbers (percentages) of bacterial species in each family. Bacterial isolates from 49 bobwhites were identified to species or genus level by 16S rRNA gene sequencing and biochemistry.

### Influence of Age, Sex, and Tissue Type on Quail Cultivable Bacterial Microbiota

Significant differences in the distribution of bacterial taxa among age, sex, and tissue groups were detected. At the genus level, the regression models explained between 16% and 57% of the variation in the distribution of the eight most common bacterial genera (Table 3). Of these eight genera, only three differed significantly between males and females (Binomial Logistic Regression, Table 3). *Enterococcus* was more common than expected in females (p = 0.013) and *Rothia* and *Streptococcus* were more common than expected in males (p = 0.024 and 0.036, respectively). Only *Paenibacillus* differed between age classes: it was more common than expected in juveniles (p = 0.05, Table 3). For *Cronobacter* the sex by age interaction term was significant: this genus was much less common than expected in adult females and slightly more common than expected in adult males (p = 0.04, Table 3). At species level, *Enterococcus faecium* was more common in females than males (p = 0.043) and *Rothia nasimurium* was more common in males than females (p = 0.026).

**Table 3:** Influence of age, sex and tissue type on the distribution of microbial taxa*.

*Family*	# of Taxa	P	R^2^	M	F	Sex-P	A	J	Age-P	Tr	Cr	Ce	Cl	Tissue-p
*Bacillus*	234	<0.0001	0.12	142	92		179	55		107	49	77	1	<0.0001
*Bifidobacterium*	14	<0.0001	0.41	9	5		12	2		13	1	0	0	<0.0001
*Cronobacter*	24	<0.0001	0.53	17	7		18	6		0	0	24	0	<0.0001
*Enterococcus*	60	<0.0001	0.22	26	34	0.013	46	14		42	12	5	1	<0.0001
*Escherichia*	14	<0.0001	0.41	8	6		12	2		13	0	1	0	<0.0001
*Lactobacillus*	51	<0.0001	0.14	28	23		37	14		17	0	29	5	<0.0001
*Paenibacillus*	53	<0.0001	0.16	27	26		33	20	0.02	27	3	23	0	<0.0001
*Rothia*	17	<0.0001	0.40	13	4	0.024	15	2		0	1	16	0	<0.0001
*Staphylococcus*	16	0.04	0.13	8	8		11	5		1	4	11	0	0.008
*Streptococcus*	60	0.0005	0.11	41	19	0.036	51	9		20	18	22	0	0.0006
*Streptomyces*	24	0.0006	0.19	16	8		19	5		1	1	20	2	<0.0001

*Data shown are total number of taxa found in the samples. P, p-value; M, Male; F, female. A, adult; J, juvenile; T, trachea; Cr, crop; Ce, cecum, Cl, cloacal. A p-value is not provided when it is greater than 0.05.

Most variations in quail microbiota were driven by tissue types or anatomical locations. Compared to intestinal microbiota, tracheal microbiota was less diverse, as evidenced by the low number of taxa found in tracheal samples. All of the 8 most common bacterial genera differed significantly among tissue types (P<0.0002 for all). *Cronobacter*, *Rothia*, and *Streptomyces* were most common in crop while *Enterococcus*, *Lactobacillus* and *Paenibacillus* were most common in cecum. *Streptococcus* and *Bacillus* were fairly common in all three sections of the GI tract but were very rare or absent from the trachea.

### Bacterial Species Diversity of Quail Cultivable Bacterial Microbiota

**Figure 1:**
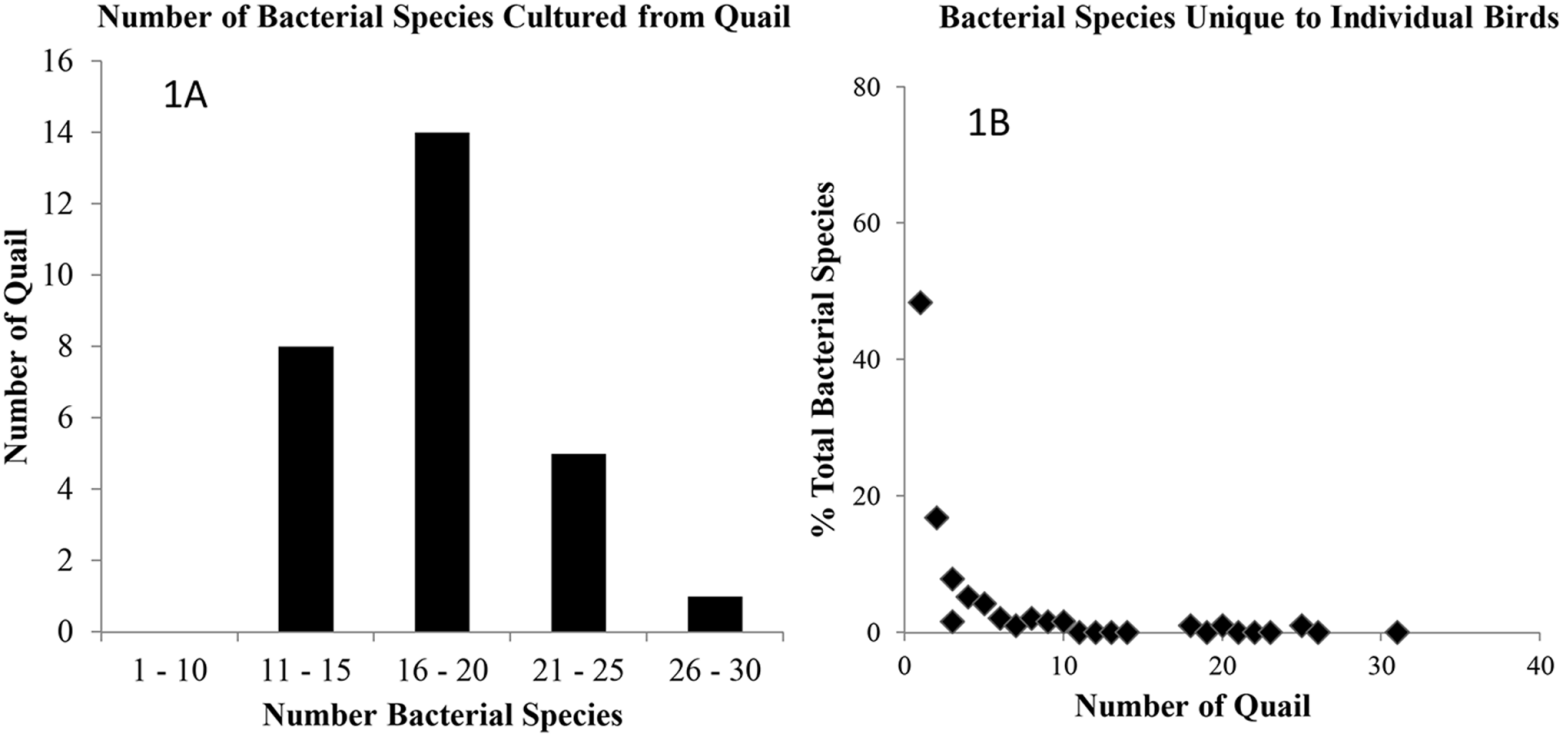
The number of bacterial species and the species diversity of northern bobwhite cultivable bacterial microbiota. 1A. Species data from 28 individual quail for which all four tissue types are presented. Cultures of most quail yielded 16 to 20 bacterial species while the cultures of a moribund quail gave rise to 26 bacterial species. 1B. Data presented are percentage of unique bacterial species (total number = 190) found in individual quail. Vast majority of bacterial species were unique to individual quail.

Data for 28 quail for which all four tissue types were available for culture were analyzed and presented in Fig. 1A. The number of bacterial taxa found per tissue analyzed did not differ significantly by sex, age or the interaction between sex and age, but did differ significantly by the type of tissue analyzed (GLM_Poisson distribution_: Overall ChiSquare = 126, DF = 6, P<0.0001, P values for sex, age and sex*age >0.1, P value for tissue type <0.0001). Cultures of most quail yielded 16 to 20 bacterial species per bird. The cultures of a moribund juvenile bird yielded 26 bacterial species (Fig. 1A). Extensive variation in the composition of quail microbiota was observed among individual birds (Fig. 1B). Approximately 48% of bacterial species were unique to individual birds; 17%, 8%, 5%, and 4% of bacterial species were found in 2, 3, 4, and 5 birds, respectively (Fig. 1B). In general, less than 3% of bacterial species could be found in more than 7 quail.

**Figure 2:**
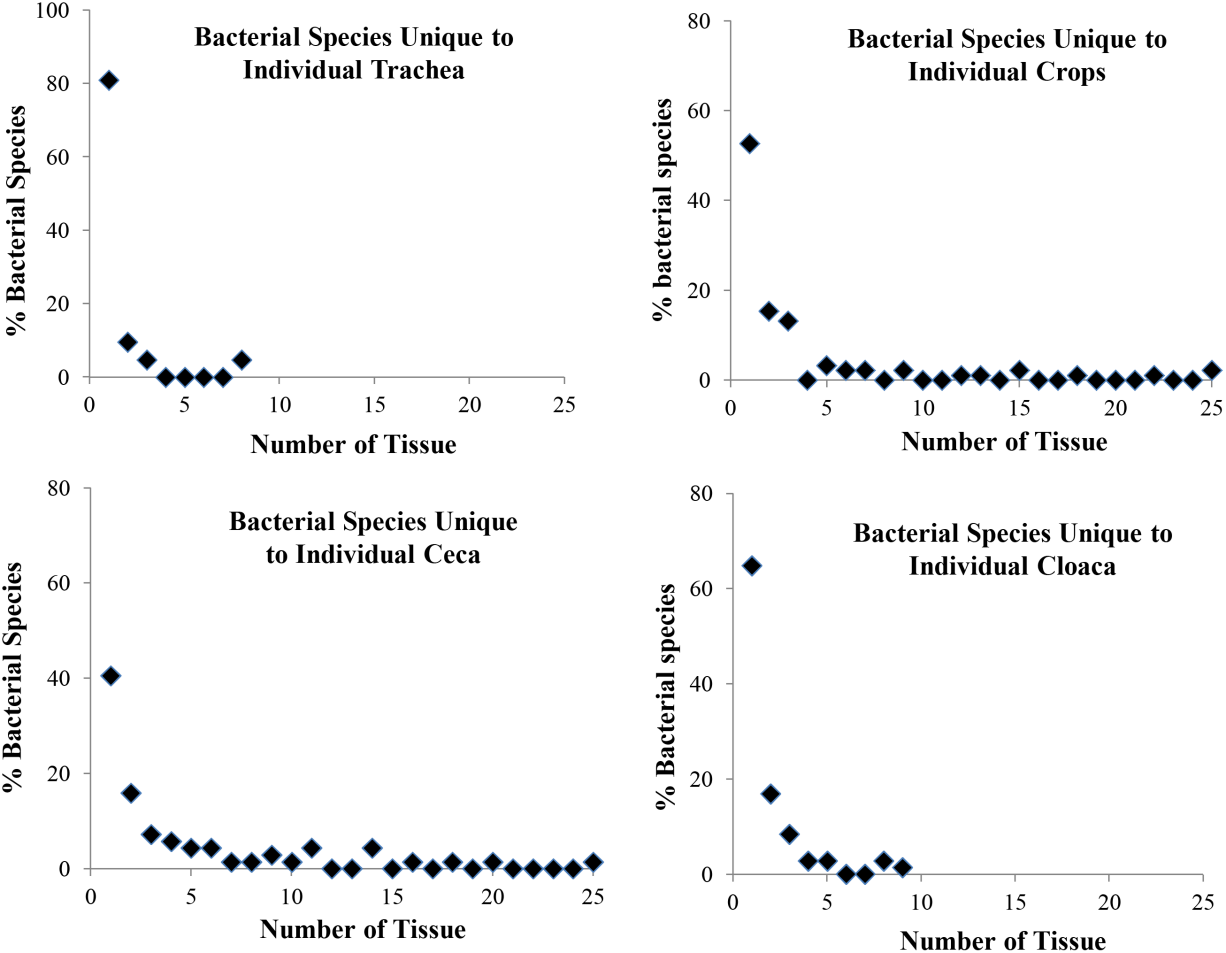
Tissue-specific species diversity of northern bobwhite cultivable bacterial microbiota. Data shown are the percentages of unique bacterial species (total number = 190) found in individual quail. Vast majority of bacterial species were unique to individual tissue samples.

At the tissue level, samples from the cecum had the highest number of bacterial species (6.25±2.89, N = 36 analyzed) followed by crop (6.4±2.78, N = 43), cloaca (4.0±0.2.67, N = 29) and trachea (1.36±0.74, N = 14). Means from all four tissue types differed significantly (Least Squares Means Student’s t test, p<0.05). Eighty one percent of tracheal bacterial species were unique to individual tissue samples; and only 10% and 5% were found in 2 and 3 tissues, respectively (Fig. 2A). About 53% of crop bacterial species were unique to individual samples and less than 4% of bacterial species were found in 4 or more samples (Figure 2B). Compared to crop microbiota, less diversity was associated with cecal microbiota. About 41% of cecal bacterial species were unique to individual samples and less than 4% of bacterial species were found in seven or more samples (Fig. 2C). The cloacal microbiota also demonstrated extensive diversity among birds with 65% of bacterial species being unique to individual samples and less than 3% of bacterial species being isolated from 4 or more samples (Fig. 2D).

**Figure 3:**
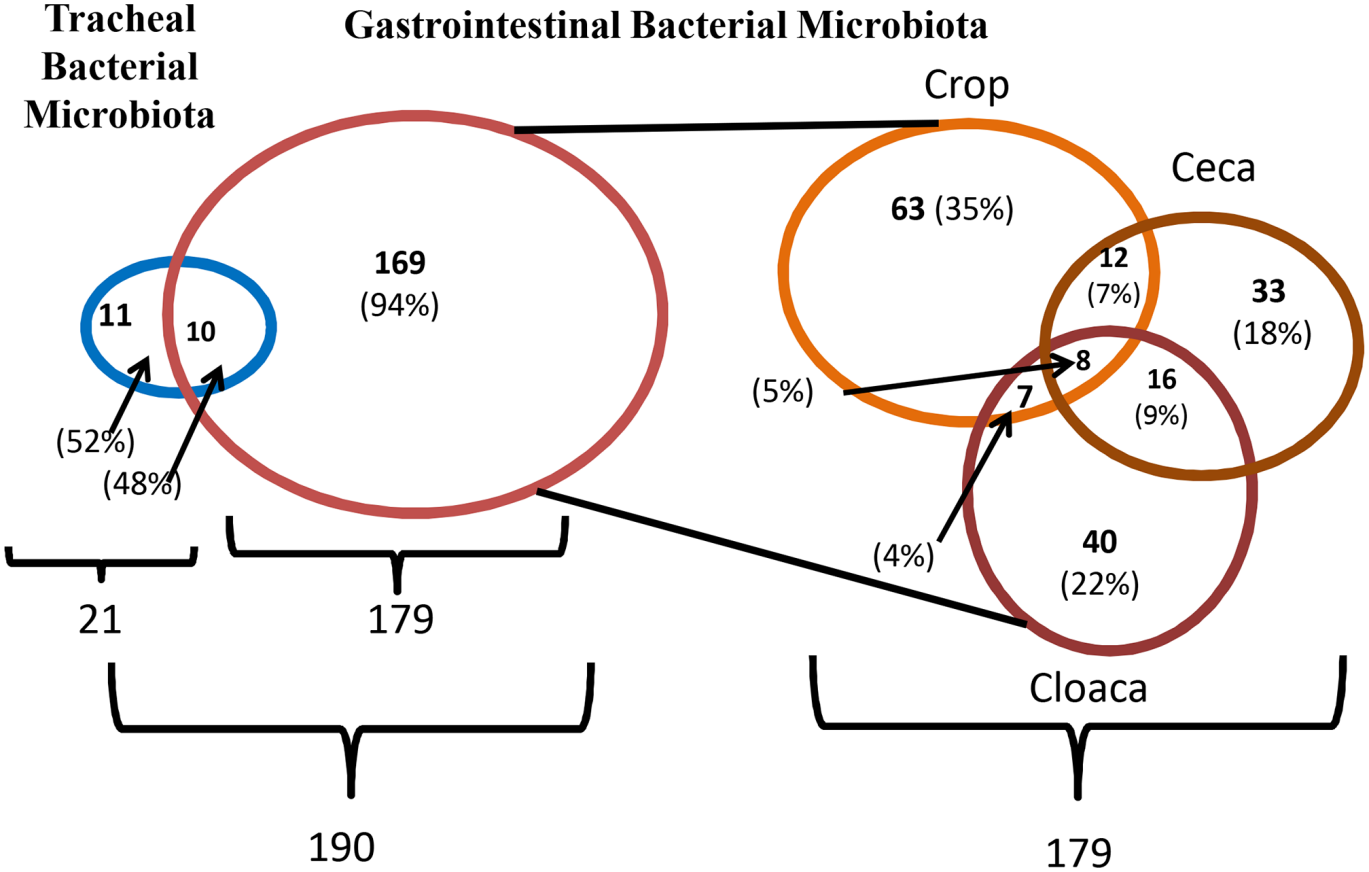
Cross-tissue species diversity of northern bobwhite microbiota. Data shown are the numbers (percentage) of species that are unique or shared by tissue-specific quail cultivable bacterial microbiota. About 47.62% tracheal bacterial were cultured from gastrointestinal tract, including *Acinetobacter baumannii*, *Bacillus aryabhattai*, *Bacillus subtilis*, *Enterococcus faecium*, *Klebsiella oxytoca*, *Lactobacillus agilis*, *Lactobacillus salivarius*, *Lactobacillus gasseri*, *Microbacterium testaceum*, and *Pseudomonas aeruginos*. Species diversity was detected throughout the gastrointestinal tract with only 8 (4.47%) common bacterial species including *Bacillus cereus, Bacillus megaterium, Bacillus nealsonii, Bacillus pumilus, Bacillus simplex, Bacillus subtilis, Enterococcus faecium,* and *Staphylococcus gallinarum*.

Cross-tissue analysis of microbiota showed that 48% of tracheal species were found in the intestine (Fig. 3). These species included *Acinetobacter baumannii*, *Bacillus aryabhattai*, *Bacillus subtilis*, *Enterococcus faecium*, *Klebsiella oxytoca*, *Lactobacillus agilis*, *Lactobacillus salivarius*, *Lactobacillus gasseri*, *Microbacterium testaceum*, and *Pseudomonas aeruginos*. Variation throughout the intestinal tract was detected. Only 8 bacterial species (4.47%) were consistently cultured from crops, ceca, and cloaca (Figure 3). The common species of intestinal microbiota included *Bacillus cereus, Bacillus megaterium, Bacillus nealsonii, Bacillus pumilus, Bacillus simplex, Bacillus subtilis, Enterococcus faecium,* and *Staphylococcus gallinarum*. *Bacillus subtilis* and *Enterococcus faecium* were the common species found in all tissue specific microbiota.

### Pathogenic and Opportunistic Pathogenic Bacterial Species

*Mycobacterium* sp., possibly a member of the *M. terrae* complex, was isolated from the trachea of one bird. *Pseudomonas aeruginosa* was identified from 12 (24%) birds, including 8 tracheae, 2 crops, and 3 ceca. *E. coli* was cultured from 16 (32%) quail, including 1 crop and 15 ceca (Table 4). In addition, several species usually considered as human commensal organisms, including *Neisseria flavescens* (6%) and *Neisseria sicca* (6%) were cultured from the ceca and cloaca of quail (Table 4).

**Table 4:** Prevalence of opportunistic pathogens in quail*.

Species	# (%) ofquail	Tissue				Sex		Age		
		Trachea	Crop	Ceca	Cloaca	Male	Female	Adult	Juvenile	Unknown
*E. coli*	16	0	1	15	0	9	7	12	3	1
	(32%)		(2.08%)	(38.46%)		(56.25%)	(43.75%)	(75%)	(18.75%)	(6.25%)
*Ps. aeruginosa*	12	8	2	3	0	5	7	11	1	0
	(24%)	(16.32%)	(4.17%)	(7.69%)		(41.67%)	(58.33%)	(91.67%)	(8.33%)	
*N. flavescens*	3	0	0	1	2	3	0	2	1	0
	(6%)			(2.56%)	(5.26%)	(100%)		(66.67%)	(33.33%)	
*N. sicca*	3	0	0	0	3	1	2	3	0	0
	(6%)				(7.89%)	(33.33%)	(66.67%)	(100%)		
*S. mitis*	9	0	0	1	8	7	2	6	3	0
	(18%)			(2.56%)	(21.05%)	(77.78%)	(22.22%)	(66.67%)	(33.33%)	

*Shown are the numbers (percentages) of quail and tissue tested positive.

### Antimicrobial Resistance of Quail Cultivable Bacterial Microbiota

Phenotypic analysis of quail bacterial isolates revealed varying degrees of resistance to multiple classes of antimicrobials (Table 5 and 6). Resistance to penicillin, clindamycin, erythromycin, and sulphadimethoxine was detected in all *E. coli* (100%) isolates (n = 16). Resistance to florfenicol and sulphatiazole were found in 75% and 25% of isolates, respectively (Table 5). Intermediate susceptibility to spectinomycin, florfenicol and sulphatiazole was shown by 100%, 75%, and 25% of *E. coli* isolates, respectively. Similar resistance patterns were detected in *Enterobacter* and *Neisseria* isolates except that most *Enterobacter* isolates were also resistant to amoxicillin and *Neisseria* isolates were susceptible to florfenicol. Resistance to 15 of the 18 antimicrobials tested was detected in vast majority of *Ps. aeruginosa* isolates (Table 5). Intermediate susceptibility to Oxytetracycline was found in 54% of the isolates. Gentamicin and Neomycin were the only 2 antimicrobials effective against all *Ps. aeruginosa* isolates. Antimicrobial resistance was also detected in Gram positive bacteria (Table 6). Most *E. faecalis* isolates were resistant to ceftiofur, streptomycin, clindamycin, sulphadimethoxine, and sulphatiazole. Intermediate susceptibility to spectinomycin and enrofloxacin was detected in *E. faecalis* isolates. *B. subtlis* and *S. gallinarum* were less resistant than *E. faecalis* to several antimicrobials tested.

**Table 5:** Antimicrobial susceptibility pattern of Gram negative bacteria isolated from bobwhites*.

	Antimicrobial	*E. coli*	*Enterobacter* sp	*Neisseria sp.*	*Ps. aeruginosa*
	agents	(n = 16)	(n = 13)	(n = 5)	(n = 13)
Beta-lactam	Amoxicillin	4–8 (100)	2–8 (31), **≥16 (69)**	0.25–0.5 (100)	**≥16 (100)**
	Ceftiofur	0.25–0.5 (100)	0.25–1 (100)	≤0.25 (100)	**≥4 (100)**
	Penicillin	**≥8 (100)**	**≥8 (100)**	0.06 (17), **0.25–1 (83)**	**≥8 (100)**
Aminoglycoside	Gentamicin	0.5–1 (100)	≤0.5 (100)	≤0.5 (100)	1–2 (100)
	Neomycin	≤2 (100)	≤2 (100)	≤2 (100)	2–8 (100)
	Spectinomycin	***16 (100)***	≤8 (33), ***16–32 (77)***	≤8 (60), **16 (40)**	**≥64 (100)**
	Streptomycin	≤8 (100)	8–32 (100)	≤8 (100)	**32 (100)**
Tetracycline	Oxytetracycline	1–2 (100)	0.25–4 (100)	0.5–1 (100)	4 (56), ***8 (54)***
	Tetracycline	1–2 (100)	0.5–4 (100)	1–2 (100)	**≥8 (100)**
Phenicol	Florfenicol	***4 (75)*** **, 8 (25)**	**1–4 (54)**	≤1 (100)	**≥8 (100)**
Macrolide	Erythromycin	**≥4 (100)**	**≥4 (100)**	**≥4 (100)**	**≥4 (100)**
	Tylosin	**≥20 (100)**	**≥20 (100)**	**≥20 (100)**	**≥20 (100)**
Lincosamide	Clindamycin	**≥4 (100)**	**≥4 (100)**	**≥4 (100)**	**≥4 (100)**
Quinolone	Enrofloxacin	≤0.12 (100)	≤0.12 (100)	0.12 (100)	***1 (85)*** **, 2 (15)**
	Novobiocin	**≥4 (100)**	**≥4 (100)**	**≥4 (100)**	**≥4 (100)**
Sulpha	Sulphadimethoxine	**≥256 (100)**	≤32 (25), **≥256 (85)**	≤32 (100)	**≥256 (100)**
	Sulphatiazole	***32–64 (25)*** **, ≥256 (75)**	***32 (25),*** **256 (85)**	≤32 (100)	**64–256 (100)**
	Trimethoprim/Sulpha	≤0.5 (100)	≤0.5 (100)	0.5–1 (100)	**≥2 (100)**

*The ranges of MICs (% of isolates) are indicated as follows: regular font, susceptible; bold and italic, intermediate susceptible; and bold and underlined, resistant.

**Table 6:** Antimicrobial susceptibility pattern of Gram positive bacteria isolated from bobwhites*.

	Antimicrobial	*B. subtilis*	*E. faecalis*	*S. gallinarum*
	Agents	(n = 10)	(n = 10)	(n = 10)
Beta-lactam	Amoxicillin	≤0.25 (100)	0.25–5 (100)	≤0.25 (100)
	Ceftiofur	≤0.25 (100)	**≥4 (100)**	0.5–1 (100)
	Penicillin	≤0.06 (100)	0.25–2 (100)	≤0.06 (100)
Aminoglycoside	Gentamicin	≤0.5 (100)	4 (70), **≥8 (30)**	≤0.5 (100)
	Neomycin	≤2 (100)	8–8 (30), **16–32 (70)**	≤2 (100)
	Spectinomycin	8 (80), ***16 (20)***	***32 (100)***	***16–32 (40),*** ** ≥64 (60)**
	Streptomycin	≤8 (100)	16–64 (100)	≤8 (100)
Tetracycline	Oxytetracycline	≤0.25 (100)	0.25–2 (100)	0.25–0.5 (90), **8 (10)**
	Tetracycline	0.25–1 (100)	0.25–2 (100)	0.25–8 (10), **8 (10)**
Phenicol	Florfenicol	≤1 (100)	1–2 (100)	1–2 (90), ***4 (10)***
Macrolide	Erythromycin	≤0.12 (100)	0.12–0.5 (100)	0.25 (0), **≥4 (10)**
	Tylosin	≤2.5 (100)	≤2.5 (100)	≤2.5 (100)
Lincosamide	Clindamycin	≤0.5 (100)	≤0.5 (20), ***2 (20),*** ** ≥4 (60)**	≤0.5 (90), **≥4 (10)**
Quinolone	Enrofloxacin	≤0.12 (100)	0.25–0.5 (80), ***1 (20)***	0.15 (100)
	Novobiocin	≤0.5 (100)	0.5–2 (90), **≥4 (10)**	**≥4 (100)**
Sulpha	Sulphadimethoxine	32 (100)	**≥256 (100)**	32 (100)
	Sulphatiazole	***32 (100)***	***64 (10),*** ** ≥256 (90)**	32 (100)
	Trimethoprim/Sulpha	≤0.5 (100)	≤0.5 (100)	≤0.5 (100)

*The ranges of MICs (% of isolates) are indicated as follows: regular font, susceptible; bold and italic, intermediate susceptible; and bold and underlined, resistant.

## Discussion

The present study provides the first inventory of cultivable bacterial microbiota of northern bobwhite. Using traditional microbiological cultures, biochemical testing, and 16S rDNA sequencing, we cultured and identified a large number of quail bacteria to species level. In total, we obtained 190 bacterial species with 21, 90, 69, and 71 species from the trachea, crop, ceca, and cloaca, respectively. The species coverage was greater than what were achieved in previous studies using culture-independent technique to characterize the cloacal microbiome of parrot (49 operational taxonomic units, OTU’s) or wild black-legged kittiwakes (64 OTUs) [15], [26]. However, culture-dependent approach made it less possible for in-depth quantitative analysis of species abundance and most likely failed to capture many bacterial species that cannot be cultured in the lab. Nonetheless, a collection of diverse live cultures made it possible for phenotypic characterization of microbes of interest.

Similar to the microbiota of other wild avian species, the microbial community of quail consists primarily of 4 phyla, including *Firmicutes*, *Actinobacteria*, *Proteobacteria,* and *Bacteroidetes* [15], [26], [27], [28]. Although both *Firmicutes* and *Bacteroidetes* are predominant phyla in the microbiome of mammals or domestic poultry, *Bacteroidetes* were rarely detected in the current study or previous studies dealing with certain bird species, such as gull and parrots [15], [26], [27]. The members of *Bacteroidetes* are known for their ability to degrade carbohydrates which are readily available to granivores, including bobwhites. This could be a result of technical limitations, such as sample storage, shipment, culture media not optimized for wild quail specimens, because we did experience difficulties in maintaining the viability of certain obligate anaerobic isolates which could affect the final results. In this study, we detected a relative richness in *Actinobacteria* and *Bacillus* which may be associated with the ground-dwelling life style of bobwhites that are in close contact with many soil bacteria. These bacterial species in quail gut may help to digest cellulose in quail diet, such as greens and fibrous seeds (e.g., Russian thistle, *Salsola kali*).

This study also shows that the composition of quail microbiota was significantly affected by age, sex, and tissue type. More juvenile than adult birds carried *Paenibacillus*, a genus of bacteria widely distributed in rhizosphere, forages, and insect larvae [29], [30]. The members of this genus are known as plant growth promoting rhixovbacteria that can act as biofertilizers and biopesticides of plant pathogens. This genus of bacteria has also been demonstrated to be inhibitors of pathogenic bacteria of animals, such as *Campylobacter jejuni* and *Clostridium botulinum* [31], [32]. The antimicrobial property of *Paenibacillus* may be utilized to promote quail gut health as being done in chickens [31]. In addition to age, sex was another factor that influenced the distribution of two closely related genera, namely *Enterococcus* and *Streptococcus*. In addition, we observed that the vast majority of bacterial species were unique to individual birds and only a small percentage of bacterial species could be found in multiple birds. Variation in bacterial species composition was also detected among tissues with only eight species being conserved throughout the intestinal tract. In this study, the experimental quail were collected from multiple ranches across the Rolling Plains ecoregion which may contribute to this high degree of variation among individual quail.

We performed several types of selective and enrichment cultures to capture pathogenic bacteria that have fastidious growth requirements. However, our results show an extremely low prevalence of traditionally known respiratory and enteric pathogens in bobwhite populations. Except the *Mycobacterium* species isolated from one bird, no other obligate and zoonotic agents were cultured from bobwhite. The low prevalence could be associated with technical limitations such as media and culture conditions were not optimized for wild quail specimens. Instead, we isolated several opportunistic pathogens, such as *E. coli*, *Enterobacter* spp, and *Ps. aeruginosa*. In general, *E. coli* is not considered part of the intestinal flora of granivorous birds and its prevalence is much higher in farm-reared and restocked birds than wild populations [33], [34]. *Ps. aeruginosa* is known as an environmental organism and airway colonization by *Ps. aeruginosa* can contribute to local inflammation and respiratory disorder [35], [36]. Additionally, bobwhite cloacal microbiota harbored several species that are usually considered human commensal organisms, including *Neisseria* species and *Streptococcus mitis*. Whether these are indigenous quail bacteria or contaminants due to human activities remains to be determined.

Antimicrobial resistance is an important issue to both wildlife conservation and public health [37]. The prevalence of antimicrobial resistance in wildlife is often unknown or underestimated. This is because many resistant bacteria are components of indigenous bacterial flora and routine diagnostic and surveillance programs do not target these organisms. In the present study, we detected a strikingly high rate of antimicrobial resistance in quail microbiota. Nearly all *E. coli, Enterobacter*, and *Neisseria* isolates were resistant to following classes of antibiotics: phenicol, macrolide, lincosamide, quinolone, and sulphate. Either intermediate susceptibility or resistance to beta-lactams and aminoglycosides were also found in these Gram negative bacterial isolates. The Gram positive bacteria, *E. faecalis* and *Staphylococcus* isolates, were resistant to beta-lactam and sulphate antibiotics, in addition to aminoglycosides. All *Ps. aeruginosa* appeared to be more resistant than environmental isolates as previously reported [37]. Wild bobwhites are not subjected to the selective pressure associated with the use of antibiotics. However, habitat pollution and the release of farm-reared bobwhites could introduce resistant organisms or resistance genes into wild quail populations. Currently, erythromycin, neomycin sulfate, penicillin G, chlortetracycline, lincomycin-spectinomycin are readily available to quail farms for prophylactic or therapeutic use. Although the exact source of antimicrobial resistance in wild quail populations remains to be investigated, human exposure to quail antimicrobial resistant microbiota should not be neglected.

In conclusion, the present study characterized the cultivable bacterial microbiota of northern bobwhite. We isolated multiple *Paenibacillus* spp. that are known to be inhibitors of pathogenic bacteria. We also obtained multiple isolates potentially representing new bacterial taxa whose complete characterization is currently underway. Overall, the prevalence of pathogenic bacteria in bobwhite populations is low. However, phenotypic characterization of selected bacterial species revealed a high prevalence of resistance to multiple classes of antimicrobial agents commonly used in avian medicine. The extent of antimicrobial resistance in quail microbiota warrants further investigation on the transmission of antibiotic resistance between wild and domestic quail populations and between bobwhites and humans.

## Supporting Information

Table S1 **Bacterial species isolated from Bobwhite quail.** The 16S rRNA Gene Sequences (∼500 bp) were subjected to homology search against GenBank database by using the BLAST search algorithm as well as MicroSeq ID 2.0 500-bp library. Accession number of the best match and percent of similarity are provided. Tissue type: Ce, cecum, Cr, crop; Cl: cloaca; and Tr: trachea.
(DOCX)

## References

[1] U.S. Department of the Interior, U.S. Fish and Wildlife Service, and U.S. Department of Commerce, U.S. Census Bureau (2013) 2011 National Survey of Fishing, Hunting, and Wildlife-Associated Recreation. U.S. Fish & Wildlife Service website. Available: http://www.census.gov/prod/2012pubs/fhw11-nat.pdf. Accessed 2014 May 28.

[2] Texas Quail Study Group (2009) Quail restoration in the Post Oak Savanna, Blackland, and Coastal Prairies. Proceedings of the 2009 Texas Quail Study Group.

[3] DeMaso SJ, Peterson MJ, Purvis JR, Silvy NJ, Cooke JL (2002) A comparison of two quail abundance indices and their relationship to quail harvest in Texas. National Quail Symposium 5: 206–212. Texas Parks and Wildlife Department, Corpus Christi, Texas. 23–27 January 2002.

[4] Urban KN, Gibson AG, Dabbert CB, Presley SM (2013) Preliminary disease surveillance in west Texas quail (Galliformes: *Odontophoridae*) populations. J. Wildl. Dis. 49: 427–431. 10.7589/2011-05-133 23568922

[5] Ferro PJ, Khan O, Vuong C, Reddy SM, LaCoste L, et al.. (2012) Avian influenza virus investigation in wild bobwhite quail from Texas. Avian Dis. (Suppl 4): S858–860. 10.1637/10197-041012-ResNote.1 23402104

[6] Terhune TM, Sisson DC, Palmer WE, Faircloth BC, Stribling HL, et al (2010) Translocation to a fragmented landscape: survival, movement, and site fidelity of Northern Bobwhites. Ecol. Appl. 20: 1040–1052. 10.1890/09-1106.1 20597288

[7] Mackie RI, Sghir A, Gaskins HR (1999) Developmental microbial ecology of the neonatal gastrointestinal tract. Am. J. Clin. Nutr. (Suppl 5): S1035–1045. 10.1093/ajcn/69.5.1035s 10232646

[8] Zoetendal EG, Collier CT, Koike S, Mackie RI, Gaskins HR (2004) Molecular ecological analysis of the gastrointestinal microbiota: a review. J. Nutr. 134: 465–472. 10.1093/jn/134.2.465 14747690

[9] Musso G, Gambino R, Cassader M (2011) Interactions between gut microbiota and host metabolism predisposing to obesity and diabetes. Annu. Rev. Med. 62: 361–380. 10.1146/annurev-med-012510-175505 21226616

[10] Kamada N, Seo SU, Chen GY, Núñez G (2013) Role of the gut microbiota in immunity and inflammatory disease. Nat. Rev. Immunol. 13: 321–335. 10.1038/nri3430 23618829

[11] Gollwitzer ES, Marsland BJ (2013) Microbiota abnormalities in inflammatory airway diseases - Potential for therapy. Pharmacol. Ther. pii: S0163-7258(13)00170-8. 10.1016/j.pharmthera.2013.08.002 23969226

[12] Pragman AA, Kim HB, Reilly CS, Wendt C, Isaacson RE (2012) The lung microbiome in moderate and severe chronic obstructive pulmonary disease. PLoS One. 7: e47305. 10.1371/journal.pone.0047305 PMC3469539 23071781

[13] Lan PT, Hayashi H, Sakamoto M, Benno Y (2002) Phylogenetic analysis of cecal microbiota in chicken by the use of 16S rDNA clone libraries. Microbiol. Immunol. 46: 371–382. 10.1111/j.1348-0421.2002.tb02709.x 12153114

[14] Collado MC, Sanz Y (2007) Characterization of the gastrointestinal mucosa-associated microbiota of pigs and chickens using culture-based and molecular methodologies. J. Food Prot. 70: 2799–2804. 10.4315/0362-028x-70.12.2799 18095433

[15] van Dongen WF, White J, Brandl HB, Moodley Y, Merkling T, et al (2013) Age-related differences in the cloacal microbiota of a wild bird species. BMC Ecol. 13: 11. 10.1186/1472-6785-13-11 PMC3668179 23531085

[16] Hidasi HW, Hidasi Neto J, Moraes DM, Linhares GF, Jayme Vde S, et al (2013) Enterobacterial detection and *Escherichia coli* antimicrobial resistance in parrots seized from the illegal wildlife trade. J Zoo Wildl Med. 44(1): 1–7. 10.1638/1042-7260-44.1.1 23505696

[17] Stedt J, Bonnedahl J, Hernandez J, McMahon BJ, Hasan B, et al.. (2014) Antibiotic resistance patterns in Escherichia coli from gulls in nine European countries. Infect Ecol Epidemiol. 10.3402/iee.v4.21565 PMC3889177 24427451

[18] Cole D, Drum DJ, Stalknecht DE, White DG, Lee MD, et al (2005) Free-living Canada geese and antimicrobial resistance. Emerg. Infect. Dis. 11: 935–938. 10.3201/eid1106.040717 PMC3367595 15963291

[19] Kozak GK, Boerlin P, Janecko N, Reid-Smith RJ, Jardine C (2009) Antimicrobial resistance in *Escherichia coli* isolates from swine and wild small mammals in the proximity of swine farms and in natural environments in Ontario, Canada. Appl. Environ. Microbiol. 75: 559–566. 10.1128/AEM.01821-08 PMC2632148 19047381

[20] Guenther S, Ewers C, Wieler LH (2011) Extended-spectrum beta-lactamases producing *E. coli* in wildlife, yet another form of environmental pollution? Front. Microbiol. 2: 246. 10.3389/fmicb.2011.00246 PMC3244693 22203818

[21] Helm JD, Hines RK, Hill JE, Caver JA (1999) Multiple drug-resistant S*almonella typhimurium* DT104 and DT104b isolated in bobwhite quail (*Colinus virginianus*). Avian Dis. 43(4): 788–91. 10611997

[22] Singh IM, Singh S, Mills-Robertson F, McMurphy MA, Applegate RD, et al (2004) Antibiotic susceptibility of *Edwardsiella hoshinae* isolated from northern bobwhite quail (*Colinus virginianus*). Vet Rec. 2004 Jul 3 155(1): 29. 10.1136/vr.155.1.29 15264489

[23] Nikkari S, Lopez L, Lepp PW, Cieslak PR, Ladd-Wilson S, et al (2002) Broad-range bacterial detection and the analysis of unexplained death and critical illness. Emerg. Infect. Dis. 8: 188–194. 10.3201/eid0802.010150 PMC2732447 11897072

[24] Janda JM, Abbott SL (2007) 16S rRNA gene sequencing for bacterial identification in the diagnostic laboratory: pluses, perils, and pitfalls. J. Clin. Microbiol 45: 2761–2764. 10.1128/JCM.01228-07 PMC2045242 17626177

[25] Clinical and Laboratory Standards Institute (2007) M100-S17. Performance standards for antimicrobial susceptibility testing; 16th informational supplement. Clinical and Laboratory Standards Institute, Wayne, PA.

[26] Xenoulis PG, Gray PL, Brightsmith D, Palculict B, Hoppes S, et al (2010) Molecular characterization of the cloacal microbiota of wild and captive parrots. Vet. Microbiol. 146: 320–325. 10.1016/j.vetmic.2010.05.024 20646876

[27] Kohl KD (2012) Diversity and function of the avian gut microbiota. J Comp Physiol B. 2012 182(5): 591–602. 10.1007/s00360-012-0645-z 22246239

[28] Waite DW, Deines P, Taylor MW (2012) Gut microbiome of the critically endangered New Zealand parrot, the kakapo (*Strigops habroptilus*). PLoS One. 7: e35803. 10.1371/journal.pone.0035803 PMC3329475 22530070

[29] El-Hadad ME, Mustafa MI, Selim ShM, El-Tayeb TS, Mahgoob AEA, et al (2011) The nematicidal effect of some bacterial biofertilizers on Meloidogyne incognita in sandy soil. Braz. J. Microbiol. 42: 105–113. 10.1590/S1517-83822011000100014 PMC3768928 24031611

[30] Wang LY, Li J, Li QX, Chen SF (2013) *Paenibacillus beijingensis* sp. nov., a nitrogen-fixing species isolated from wheat rhizosphere soil. Antonie. Van. Leeuwenhoek. 104: 675–683. 10.1007/s10482-013-9974-5 23912443

[31] Stern NJ, Svetoch EA, Eruslanov BV, Kovalev YN, Volodina LI, et al (2005) *Paenibacillus polymyxa* purified bacteriocin to control *Campylobacter jejuni* in chickens. J. Food Prot. 68: 1450–1453. 10.4315/0362-028x-68.7.1450 16013385

[32] Girardin H, Albagnac C, Dargaignaratz C, Nguyen-The C, Carlin F (2002) Antimicrobial activity of foodborne *Paenibacillus* and *Bacillus* spp. against *Clostridium botulinum*. J. Food Prot. 65: 806–813. 10.4315/0362-028x-65.5.806 12030292

[33] Glünder G (2002) Influence of diet on the occurrence of some bacteria in the intestinal flora of wild and pet birds. Dtsch. Tierarztl. Wochenschr. 109: 266–270. 12125172

[34] Díaz-Sánchez S, Moriones AM, Casas F, Höfle U (2012) Prevalence of *Escherichia coli*, *Salmonella* sp. and *Campylobacter* sp. in the intestinal flora of farm-reared, restocked and wild red-legged partridges (*Alectoris rufa*): is restocking using farm-reared birds a risk? Eur. J. Wildl. Res. 58: 99–105.

[35] Williams HD, Davies JC (2012) Basic science for the chest physician: *Pseudomonas aeruginosa* and the cystic fibrosis airway. Thorax. 67: 465–467. 10.1136/thoraxjnl-2011-201498 22382597

[36] Markarian M (1975) *Pseudomonas aeruginosa*–the causative agent of infection in birds. Vet. Med. Nauki. 12: 33–39. 127412

[37] Lutz JK, Lee J (2011) Prevalence and antimicrobial-resistance of *Pseudomonas aeruginosa* in swimming pools and hot tubs. Int. J. Environ. Res. Public Health. 8: 554–564. 10.3390/ijerph8020554 PMC3084478 21556203

