# Supplementary material for: Cultivable Bacterial Microbiota of Northern Bobwhite (Colinus virginianus): A New Reservoir of Antimicrobial Resistance?
Source: PLoS One. 2014 Jun 17;9(6):e99826. doi: 10.1371/journal.pone.0099826 (PMC4061065; doi:10.1371/journal.pone.0099826)
Supplement: Table S1 — Bacterial species isolated from Bobwhite quail. The 16S rRNA Gene Sequences (∼500 bp) were subjected to homology search against GenBank database by using the BLAST search algorithm as well as MicroSeq ID 2.0 500-bp library. Accession number of the best match and percent of similarity are provided. Tissue type: Ce, cecum, Cr, crop; Cl: cloaca; and Tr: trachea. (DOCX) [file pone.0099826.s001.docx]

Table S1. Bacterial species isolated from Bobwhite quail.*

| **Description** | **Accession** | [**Max Ident**](http://blast.ncbi.nlm.nih.gov/Blast.cgi?CMD=Get&ALIGNMENTS=100&ALIGNMENT_VIEW=Pairwise&DATABASE_SORT=0&DESCRIPTIONS=100&ENTREZ_QUERY=all%20%5bfilter%5d%20NOT(environmental%20samples%5bfilter%5d%20OR%20metagenomes%5borgn%5d)&FIRST_QUERY_NUM=0&FORMAT_OBJECT=Alignment&FORMAT_PAGE_TARGET=&FORMAT_TYPE=HTML&GET_SEQUENCE=yes&I_THRESH=&MASK_CHAR=2&MASK_COLOR=1&NEW_VIEW=yes&NUM_OVERVIEW=100&OLD_BLAST=false&PAGE=MegaBlast&QUERY_INDEX=0&QUERY_NUMBER=0&RESULTS_PAGE_TARGET=&RID=K2HX1X3C01R&SHOW_LINKOUT=yes&SHOW_OVERVIEW=yes&STEP_NUMBER=&DISPLAY_SORT=3&HSP_SORT=3#sort_mark)**ity** | **Tissue** |
| --- | --- | --- | --- |
| *Achromobacter spanius* | KF150361.1 | ≥99% | Cr |
| *Acinetobacter baumannii* | [CP001937.1](http://www.ncbi.nlm.nih.gov/nucleotide/347591739?report=genbank&log$=nucltop&blast_rank=1&RID=RBKBBSJK012) | ≥99% | Cr, Tr |
| *Acinetobacter calcoaceticus* | [JN934383.1](http://www.ncbi.nlm.nih.gov/nucleotide/379047188?report=genbank&log$=nucltop&blast_rank=1&RID=NHXX4W0M013) | ≥99% | Ce, Cr |
| *Acinetobacter genomosp.* | [FJ860875.1](http://www.ncbi.nlm.nih.gov/nucleotide/227121246?report=genbank&log$=nucltop&blast_rank=4&RID=WFNN5272013) | ≥99% | Ce, Cr |
| *Acinetobacter radioresistens* | [JN669165.1](http://www.ncbi.nlm.nih.gov/nucleotide/364536730?report=genbank&log$=nucltop&blast_rank=1&RID=WFPN5AD301N) | ≥99% | Cr |
| *Anaerococcus vaginalis* | [AB691575.1](http://www.ncbi.nlm.nih.gov/nucleotide/371919675?report=genbank&log$=nucltop&blast_rank=1&RID=KXRCZ42H01R) | ≥99% | Cl |
| *Arthrobacter globiformis* | [EU221365.1](http://www.ncbi.nlm.nih.gov/nucleotide/161172558?report=genbank&log$=nucltop&blast_rank=1&RID=KXM6B6DG01R) | ≥99% | Cl, Cr |
| *Arthrobacter koreensis* | [JQ282805.1](http://www.ncbi.nlm.nih.gov/nucleotide/374719763?report=genbank&log$=nucltop&blast_rank=1&RID=NHVY5TW5012) | ≥99% | Ce |
| *Arthrobacter luteolus* | [JQ218454.1](http://www.ncbi.nlm.nih.gov/nucleotide/373943310?report=genbank&log$=nucltop&blast_rank=1&RID=KXN61TYM01R) | ≥99% | Cl |
| *Bacillus amyloliquefaciens* | [JQ696830.1](http://www.ncbi.nlm.nih.gov/nucleotide/387935327?report=genbank&log$=nucltop&blast_rank=2&RID=VWJ4G7C401S) | ≥99% | Ce, Cr |
| *Bacillus anthracis* | [JQ282824.1](http://www.ncbi.nlm.nih.gov/nucleotide/374719782?report=genbank&log$=nucltop&blast_rank=1&RID=KXP10XMF01R) | ≥99% | Cl, Ce |
| *Bacillus aryabhattai* | [JQ312015.1](http://www.ncbi.nlm.nih.gov/nucleotide/377830670?report=genbank&log$=nucltop&blast_rank=1&RID=NHXFT9AP012) | ≥99% | Ce, Cr, Tr |
| *Bacillus atrophaeus* | [JN824998.1](http://www.ncbi.nlm.nih.gov/nucleotide/373427922?report=genbank&log$=nucltop&blast_rank=2&RID=NHUD9S45012) | ≥99% | Ce |
| *Bacillus bataviensis* | EU334358.1 | ≥99% | Cr |
| *Bacillus boroniphilus* | [JN867119.1](http://www.ncbi.nlm.nih.gov/nucleotide/359392732?report=genbank&log$=nucltop&blast_rank=1&RID=NHXVKUPT013) | ≥99% | Ce |
| *Bacillus carboniphilus* | [JN867119.1](http://www.ncbi.nlm.nih.gov/nucleotide/359392732?report=genbank&log$=nucltop&blast_rank=1&RID=NHXVKUPT013) | ≥99% | Cl |
| *Bacillus cereus* | [JQ282814.1](http://www.ncbi.nlm.nih.gov/nucleotide/374719772?report=genbank&log$=nucltop&blast_rank=1&RID=NHX3B8BZ016) | ≥99% | Cl, Ce, Cr |
| *Bacillus circulans* | [JQ420892.1](http://www.ncbi.nlm.nih.gov/nucleotide/375151843?report=genbank&log$=nucltop&blast_rank=1&RID=VX262FBT01N) | ≥99% | Cr |
| *Bacillus endophyticus* | [EU221417.1](http://www.ncbi.nlm.nih.gov/nucleotide/161172610?report=genbank&log$=nucltop&blast_rank=1&RID=KXMYD73A01R) | ≥99% | Cl |
| *Bacillus infantis* | [JQ312662.1](http://www.ncbi.nlm.nih.gov/nucleotide/374722909?report=genbank&log$=nucltop&blast_rank=1&RID=NHXKR3JR012) | ≥99% | Ce |
| *Bacillus koreensis* | [JF819709.1](http://www.ncbi.nlm.nih.gov/nucleotide/334980907?report=genbank&log$=nucltop&blast_rank=2&RID=NHXU2JE9012) | ≥99% | Ce |
| *Bacillus licheniformis* | [AY647288.1](http://www.ncbi.nlm.nih.gov/nucleotide/50980353?report=genbank&log$=nucltop&blast_rank=1&RID=KXPRAFWC01S) | ≥99% | Cl |
| *Bacillus litoralis* | JN941299.1 | ≥99% | Cl |
| *Bacillus macroides* | [AJ491708.1](http://www.ncbi.nlm.nih.gov/nucleotide/21665860?report=genbank&log$=nucltop&blast_rank=1&RID=NHW7B1H0013) | ≥99% | Ce |
| *Bacillus megaterium* | JN411397.1 | ≥99% | Cl, Ce, Cr |
| *Bacillus methylotrophicus* | [JN700158.1](http://www.ncbi.nlm.nih.gov/nucleotide/375004774?report=genbank&log$=nucltop&blast_rank=1&RID=KXKWA38101S) | ≥99% | Cl |
| *Bacillus muralis* | [HE646789.1](http://www.ncbi.nlm.nih.gov/nucleotide/375339399?report=genbank&log$=nucltop&blast_rank=3&RID=NHUB9EHD013) | ≥99% | Ce |
| *Bacillus mycoides* | [HM224388.1](http://www.ncbi.nlm.nih.gov/nucleotide/297533415?report=genbank&log$=nucltop&blast_rank=5&RID=VWHRUGBP01S) | ≥99% | Cr |
| *Bacillus nealsonii* | JN644556.1 | ≥99% | Cl, Ce, Cr |
| *Bacillus niacini* | JN993716.1 | ≥99% | Cl, Cr |
| *Bacillus odysseyi* | [AB681147.1](http://www.ncbi.nlm.nih.gov/nucleotide/359805212?report=genbank&log$=nucltop&blast_rank=1&RID=VWJN9ATC01S) | ≥99% | Cr |
| *Bacillus pumilus* | JQ046373.1 | ≥99% | Cl, Ce, Cr |
| *Bacillus safensis* | [AB697713.1](http://www.ncbi.nlm.nih.gov/nucleotide/375268449?report=genbank&log$=nucltop&blast_rank=1&RID=NHWHSHDR016) | ≥99% | Cl, Ce |
| *Bacillus selenatarsenatis* | [HQ202857.1](http://www.ncbi.nlm.nih.gov/nucleotide/309253939?report=genbank&log$=nucltop&blast_rank=4&RID=KXPKP1BB01R) | ≥99% | Cl |
| *Bacillus simplex* | [JN544152.1](http://www.ncbi.nlm.nih.gov/nucleotide/345848038?report=genbank&log$=nucltop&blast_rank=3&RID=WFN3NBD2012) | ≥99% | Cl, Ce, Cr |
| *Bacillus soli* | [HQ406769.1](http://www.ncbi.nlm.nih.gov/nucleotide/312179368?report=genbank&log$=nucltop&blast_rank=4&RID=WFPVG0BJ01S) | ≥99% | Cr |
| *Bacillus subtilis* | JN644613.1 | ≥99% | Cl, Ce, Cr, Tr |
| *Bacillus thuringiensis* | [JQ621963.1](http://www.ncbi.nlm.nih.gov/nucleotide/378835187?report=genbank&log$=nucltop&blast_rank=3&RID=NHX4SFP801N) | ≥99% | Cl, Ce |
| *Bacillus vallismortis* | [JN112317.1](http://www.ncbi.nlm.nih.gov/nucleotide/344944439?report=genbank&log$=nucltop&blast_rank=1&RID=VX3035N901S) | ≥99% | Cr |
| *Bacillus vietnamensis* | [AB697709.1](http://www.ncbi.nlm.nih.gov/nucleotide/375268445?report=genbank&log$=nucltop&blast_rank=1&RID=NHXNHN2701N) | ≥99% | Ce |
| *Bifidobacterium saeculare* | AB507153.1 | ≥99% | Cl, Ce |
| *Brevibacillus brevis* | [AM292059.1](http://www.ncbi.nlm.nih.gov/nucleotide/161513480?report=genbank&log$=nucltop&blast_rank=2&RID=VWJUUPDX01N) | ≥99% | Cr |
| *Cellulosimicrobium funkei* | [AY729960.1](http://www.ncbi.nlm.nih.gov/nucleotide/52430436?report=genbank&log$=nucltop&blast_rank=4&RID=VX37SMZE01N) | ≥99% | Cr |
| *Clostridium sordellii* | [JN048960.1](http://www.ncbi.nlm.nih.gov/nucleotide/357435454?report=genbank&log$=nucltop&blast_rank=2&RID=KXRNVJWT01S) | ≥99% | Cl, Ce |
| *Corynebacterium alkanolyticum* | [AB595210.1](http://www.ncbi.nlm.nih.gov/nucleotide/329790847?report=genbank&log$=nucltop&blast_rank=1&RID=VWHNFSH301S) | ≥99% | Cr |
| *Corynebacterium confusum* | EU725778.1 | ≥99% | Cl |
| *Cronobacter sakazakii* | [GU122184.1](http://www.ncbi.nlm.nih.gov/nucleotide/268527638?report=genbank&log$=nucltop&blast_rank=1&RID=VWK38ADS01S) | ≥99% | Cr |
| *Cronobacter turicensis* | [FN543093.2](http://www.ncbi.nlm.nih.gov/nucleotide/323575285?report=genbank&log$=nucltop&blast_rank=1&RID=WFP74D5J013) | ≥99% | Cr |
| *Enterobacter asburiae* | [JQ682630.1](http://www.ncbi.nlm.nih.gov/nucleotide/383793418?report=genbank&log$=nucltop&blast_rank=2&RID=VX1DN9FA01N) | ≥99% | Cr |
| *Enterobacter cloacae* | [GU186117.1](http://www.ncbi.nlm.nih.gov/nucleotide/270300973?report=genbank&log$=nucltop&blast_rank=4&RID=VX1H18GZ01N) | ≥99% | Cr |
| *Enterobacter hormaechei* | [JN835523.1](http://www.ncbi.nlm.nih.gov/nucleotide/377657262?report=genbank&log$=nucltop&blast_rank=2&RID=NHTXVZCE016) | ≥99% | Ce |
| *Enterococcus casseliflavus* | [AB699730.1](http://www.ncbi.nlm.nih.gov/nucleotide/378786264?report=genbank&log$=nucltop&blast_rank=1&RID=NCRPGHJK01N) | ≥99% | Ce, Cr |
| *Enterococcus cecorum* | JQ297563.1 | ≥99% | Ce |
| *Enterococcus durans* | JQ266299.1 | ≥99% | Cl, Ce |
| *Enterococcus faecalis* | [AB690253.1](http://www.ncbi.nlm.nih.gov/nucleotide/365799382?report=genbank&log$=nucltop&blast_rank=1&RID=KXP2FK2N01S) | ≥99% | Cl, Ce |
| *Enterococcus faecium* | [JQ366084.1](http://www.ncbi.nlm.nih.gov/nucleotide/378947517?report=genbank&log$=nucltop&blast_rank=1&RID=NHX1X1YA013) | ≥99% | Cl, Ce, Cr, Tr |
| *Enterococcus gallinarum* | [JN859535.1](http://www.ncbi.nlm.nih.gov/nucleotide/359466014?report=genbank&log$=nucltop&blast_rank=1&RID=NCRB917T01S) | ≥99% | Ce |
| *Enterococcus hirae* | JN644509.1 | ≥99% | Cl, Ce |
| *Erwinia persicina* | [DQ122337.1](http://www.ncbi.nlm.nih.gov/nucleotide/71493075?report=genbank&log$=nucltop&blast_rank=1&RID=VX3TPBRC01S) | ≥99% | Cr |
| *Escherichia coli* | [JN578647.1](http://www.ncbi.nlm.nih.gov/nucleotide/347439017?report=genbank&log$=nucltop&blast_rank=1&RID=NHWBARBG016) | ≥99% | Ce, Cr |
| *Exiguobacterium acetylicum* | [JQ979082.1](http://www.ncbi.nlm.nih.gov/nucleotide/387571513?report=genbank&log$=nucltop&blast_rank=1&RID=VX0WZJJY01N) | ≥99% | Cr |
| *Gemella sanguinis* | GU561409.1 | ≥99% | Cl |
| *Gordonia lacunae* | [GU727686.1](http://www.ncbi.nlm.nih.gov/nucleotide/291061266?report=genbank&log$=nucltop&blast_rank=2&RID=WFN7VVE9012) | ≥99% | Cr |
| *Klebsiella oxytoca* | [JF317350.1](http://www.ncbi.nlm.nih.gov/nucleotide/326787470?report=genbank&log$=nucltop&blast_rank=4&RID=NHVH1JKJ01N) | ≥99% | Ce, Tr |
| *Klebsiella pneumoniae* | [HQ622344.1](http://www.ncbi.nlm.nih.gov/nucleotide/357973840?report=genbank&log$=nucltop&blast_rank=1&RID=RBKCSBYN016) | ≥99% | Tr |
| *Lactobacillus agilis* | [AB425914.1](http://www.ncbi.nlm.nih.gov/nucleotide/282154586?report=genbank&log$=nucltop&blast_rank=1&RID=NHTU7YZE013) | ≥99% | Ce, Tr |
| *Lactobacillus coleohominis* | [NR_042436.1](http://www.ncbi.nlm.nih.gov/nucleotide/343201710?report=genbank&log$=nucltop&blast_rank=1&RID=WFTP3G0Z016) | ≥99% | Cr |
| *Lactobacillus helveticus* | [HM218196.1](http://www.ncbi.nlm.nih.gov/nucleotide/306999765?report=genbank&log$=nucltop&blast_rank=4&RID=RBJJ6BDC01S) | ≥99% | Tr |
| *Lactobacillus saerimneri* | [NR_029085.1](http://www.ncbi.nlm.nih.gov/nucleotide/265678780?report=genbank&log$=nucltop&blast_rank=2&RID=VX425J6601S) | ≥99% | Cr |
| *Lactobacillus salivarius* | [CP002034.1](http://www.ncbi.nlm.nih.gov/nucleotide/300213939?report=genbank&log$=nucltop&blast_rank=1&RID=RBK3MYBS013) | ≥99% | Ce, Cr, Tr |
| *Lactococcus garvieae* | JF831159.1 | ≥99% | Cl, Ce |
| *Leclercia adecarboxylata* | [AB681872.1](http://www.ncbi.nlm.nih.gov/nucleotide/359805793?report=genbank&log$=nucltop&blast_rank=1&RID=WFNK3XBZ016) | ≥99% | Cr |
| *Leifsonia xyli* | [HQ530514.1](http://www.ncbi.nlm.nih.gov/nucleotide/313770958?report=genbank&log$=nucltop&blast_rank=1&RID=RBK6FCWP01S) | ≥99% | Tr |
| *Lysinibacillus fusiformis* | [JQ030910.1](http://www.ncbi.nlm.nih.gov/nucleotide/374095088?report=genbank&log$=nucltop&blast_rank=1&RID=KXR0819001R) | ≥99% | Cl |
| *Lysinibacillus sphaericus* | [HQ336310.1](http://www.ncbi.nlm.nih.gov/nucleotide/317159840?report=genbank&log$=nucltop&blast_rank=3&RID=NHW9HV9J01N) | ≥99% | Ce |
| *Lysinibacillus xylanilyticus* | [AB662958.1](http://www.ncbi.nlm.nih.gov/nucleotide/343480676?report=genbank&log$=nucltop&blast_rank=1&RID=NHX7S43P01S) | ≥99% | Ce |
| *Microbacterium arborescens* | JN099789.1 | ≥99% | Cl |
| *Microbacterium imperiale* | [DQ122279.1](http://www.ncbi.nlm.nih.gov/nucleotide/71493017?report=genbank&log$=nucltop&blast_rank=1&RID=VWJKNP9901S) | ≥99% | Cr |
| *Microbacterium luteolum* | [JQ282808.1](http://www.ncbi.nlm.nih.gov/nucleotide/374719766?report=genbank&log$=nucltop&blast_rank=2&RID=RBK22631016) | ≥99% | Tr |
| *Microbacterium paludicola* | [HF585381.1](http://www.ncbi.nlm.nih.gov/nucleotide/451170770?report=genbank&log$=nucltop&blast_rank=1&RID=1527CMCA01N) | ≥99% | Ce |
| *Microbacterium paraoxydans* | [AJ581908.1](http://www.ncbi.nlm.nih.gov/nucleotide/34481745?report=genbank&log$=nucltop&blast_rank=7&RID=KXN4HF9B01R) | ≥99% | Cl, Cr |
| *Microbacterium testaceum* | [AF474330.1](http://www.ncbi.nlm.nih.gov/nucleotide/20799376?report=genbank&log$=nucltop&blast_rank=2&RID=KXPVW9M401S) | ≥99% | Cl, Tr |
| *Micrococcus luteus* | JN411682.1 | ≥99% | Cl, Ce |
| *Neisseria flavescens* | GU417618.1 | ≥99% | Cl, Ce |
| *Neisseria meningitidis* | FJ932762.1 | ≥99% | Cl |
| *Neisseria sicca* | GU427244.1 | ≥99% | Cl |
| *Paenibacillus amylolyticus* | [AB115960.1](http://www.ncbi.nlm.nih.gov/nucleotide/33354183?report=genbank&log$=nucltop&blast_rank=9&RID=NHW5CCJ8016) | ≥99% | Ce |
| *Paenibacillus chibensis* | [JQ659795.1](http://www.ncbi.nlm.nih.gov/nucleotide/381217434?report=genbank&log$=nucltop&blast_rank=2&RID=VX142F2F01S) | ≥99% | Cl, Cr |
| *Paenibacillus cineris* | [JN592451.1](http://www.ncbi.nlm.nih.gov/nucleotide/371927114?report=genbank&log$=nucltop&blast_rank=4&RID=NHMMN8XV01N) | ≥99% | Ce, Cr |
| *Paenibacillus durus* | [JQ236815.1](http://www.ncbi.nlm.nih.gov/nucleotide/377830136?report=genbank&log$=nucltop&blast_rank=1&RID=NHMJKG5901S) | ≥99% | Ce |
| *Paenibacillus glycanilyticus* | [JQ647886.1](http://www.ncbi.nlm.nih.gov/nucleotide/380864335?report=genbank&log$=nucltop&blast_rank=2&RID=WFMWMEP7016) | ≥99% | Cr |
| *Paenibacillus illinoisensis* | [JQ579623.1](http://www.ncbi.nlm.nih.gov/nucleotide/380450418?report=genbank&log$=nucltop&blast_rank=3&RID=WFNXGKBM016) | ≥99% | Cr |
| *Paenibacillus jamilae* | FN649433.1 | ≥99% | Cl |
| *Paenibacillus lactis* | [JN650284.1](http://www.ncbi.nlm.nih.gov/nucleotide/375004855?report=genbank&log$=nucltop&blast_rank=2&RID=NHMEKVUA012) | ≥99% | Ce, Cr |
| *Paenibacillus larvae* | [AB680856.1](http://www.ncbi.nlm.nih.gov/nucleotide/359803907?report=genbank&log$=nucltop&blast_rank=1&RID=VX1WUP2N01S) | ≥99% | Cr |
| *Paenibacillus lautus* | [JQ029711.1](http://www.ncbi.nlm.nih.gov/nucleotide/372864083?report=genbank&log$=nucltop&blast_rank=1&RID=NHWS6PH6013) | ≥99% | Ce |
| *Paenibacillus phyllosphaerae* | [NR_043008.1](http://www.ncbi.nlm.nih.gov/nucleotide/343202627?report=genbank&log$=nucltop&blast_rank=1&RID=RBJYAXYU016) | ≥99% | Tr |
| *Paenibacillus popilliae* | [EU982938.1](http://www.ncbi.nlm.nih.gov/nucleotide/198444462?report=genbank&log$=nucltop&blast_rank=2&RID=VX1S0K2G01S) | ≥99% | Cr |
| *Paenibacillus woosongensis* | [JQ248575.1](http://www.ncbi.nlm.nih.gov/nucleotide/378966891?report=genbank&log$=nucltop&blast_rank=1&RID=NHVBUPUD016) | ≥99% | Ce |
| *Paenibacillus xylanilyticus* | [JQ649398.1](http://www.ncbi.nlm.nih.gov/nucleotide/375270234?report=genbank&log$=nucltop&blast_rank=2&RID=VX1V51A801N) | ≥99% | Cr |
| *Pantoea agglomerans* | [JN680235.1](http://www.ncbi.nlm.nih.gov/nucleotide/364521045?report=genbank&log$=nucltop&blast_rank=2&RID=NHVN6XRW012) | ≥99% | Ce, Cr |
| *Pantoea ananatis* | [GU339282.1](http://www.ncbi.nlm.nih.gov/nucleotide/285206753?report=genbank&log$=nucltop&blast_rank=2&RID=VX4477BF01N) | ≥99% | Cr |
| *Pantoea dispersa* | HQ683771.1 | ≥99% | Cr |
| *Pantoea stewartii* | [JQ660286.1](http://www.ncbi.nlm.nih.gov/nucleotide/381217925?report=genbank&log$=nucltop&blast_rank=1&RID=VX2G0V2R01N) | ≥99% | Cr |
| *Pantoea vagans* | [CP002206.1](http://www.ncbi.nlm.nih.gov/nucleotide/308056051?report=genbank&log$=nucltop&blast_rank=3&RID=WFPA0Z1G013) | ≥99% | Cr |
| *Pediococcus acidilactici* | [JN836485.1](http://www.ncbi.nlm.nih.gov/nucleotide/374722731?report=genbank&log$=nucltop&blast_rank=1&RID=KXNVFA0K01R) | ≥99% | Cl |
| *Pseudomonas aeruginosa* | [JQ267797.1](http://www.ncbi.nlm.nih.gov/nucleotide/372468023?report=genbank&log$=nucltop&blast_rank=2&RID=RBKM4ZUC016) | ≥99% | Ce, Cr, Tr |
| *Rathayibacter rathayi* | [DQ363143.1](http://www.ncbi.nlm.nih.gov/nucleotide/88909582?report=genbank&log$=nucltop&blast_rank=3&RID=WFRC418U012) | ≥99% | Cr |
| *Rhodococcus coprophilus* | [AJ298938.1](http://www.ncbi.nlm.nih.gov/nucleotide/10185118?report=genbank&log$=nucltop&blast_rank=1&RID=WFRFRSCG012) | ≥99% | Cr |
| *Rhodococcus rhodochrous* | [AB562467.1](http://www.ncbi.nlm.nih.gov/nucleotide/296841000?report=genbank&log$=nucltop&blast_rank=2&RID=WFRE5YPZ016) | ≥99% | Cr |
| *Rothia mucilaginosa* | [AM779074.1](http://www.ncbi.nlm.nih.gov/nucleotide/158147910?report=genbank&log$=nucltop&blast_rank=1&RID=KXM80GN601R) | ≥99% | Cl |
| *Rothia nasimurium* | [NR_025310.1](http://www.ncbi.nlm.nih.gov/nucleotide/219878171?report=genbank&log$=nucltop&blast_rank=1&RID=VWHDY6FE01N) | ≥99% | Cr |
| *Saccharothrix espanaensis* | NR_102474.1 | ≥99% | Cl |
| *Shigela sonnei* | EU723822.1 | ≥99% | Ce |
| *Sodalis glossinidius* | [AM237373.1](http://www.ncbi.nlm.nih.gov/nucleotide/90818776?report=genbank&log$=nucltop&blast_rank=1&RID=WFP5TM45016) | ≥99% | Cr |
| *Staphylococcus aureus* | [HE681097.1](http://www.ncbi.nlm.nih.gov/nucleotide/385195117?report=genbank&log$=nucltop&blast_rank=1&RID=WFPD2H58016) | ≥99% | Cr |
| *Staphylococcus gallinarum* | [EU046490.1](http://www.ncbi.nlm.nih.gov/nucleotide/157932969?report=genbank&log$=nucltop&blast_rank=9&RID=NHU7SPBG013) | ≥99% | Cl, Ce, Cr |
| *Staphylococcus sciuri* | JF513127.1 | ≥99% | Cl, Cr |
| *Staphylococcus warneri* | [JN644590.1](http://www.ncbi.nlm.nih.gov/nucleotide/348161502?report=genbank&log$=nucltop&blast_rank=1&RID=KXNH13BY01R) | ≥99% | Cl |
| *Stenotrophomonas maltophilia* | [JQ266297.1](http://www.ncbi.nlm.nih.gov/nucleotide/374634895?report=genbank&log$=nucltop&blast_rank=2&RID=NHWY0Z1E013) | ≥99% | Ce |
| *Streptococcus cristatus* | AY281090.1 | ≥99% | Cl, Ce |
| *Streptococcus gordonii* | GU418444.1 | ≥99% | Cl |
| *Streptococcus infantis* | [GU419740.1](http://www.ncbi.nlm.nih.gov/nucleotide/285183622?report=genbank&log$=nucltop&blast_rank=3&RID=KXMJG13S01R) | ≥99% | Cl |
| *Streptococcus mitis* | AB682354.1 | ≥99% | Cl, Ce |
| *Streptococcus pluranimalium* | [EF151155.1](http://www.ncbi.nlm.nih.gov/nucleotide/120568240?report=genbank&log$=nucltop&blast_rank=1&RID=VWJ9DZYP01S) | ≥99% | Cr |
| *Streptococcus salivarius* | FR873482.1 | ≥99% | Cl |
| *Streptococcus sanguinis* | JF803499.1 | ≥99% | Cl |
| *Streptomyces antibioticus* | [EU841627.1](http://www.ncbi.nlm.nih.gov/nucleotide/194359895?report=genbank&log$=nucltop&blast_rank=3&RID=VX40M4PX01N) | ≥99% | Cr |
| *Streptomyces bikiniensis* | [EU741193.1](http://www.ncbi.nlm.nih.gov/nucleotide/206581539?report=genbank&log$=nucltop&blast_rank=1&RID=RBJTD5MJ016) | ≥99% | Tr |
| *Streptomyces bungoensis* | [AY999905.1](http://www.ncbi.nlm.nih.gov/nucleotide/66379471?report=genbank&log$=nucltop&blast_rank=2&RID=WFPEAP4H012) | ≥99% | Cr |
| *Streptomyces calvus* | [GU433227.1](http://www.ncbi.nlm.nih.gov/nucleotide/288965936?report=genbank&log$=nucltop&blast_rank=1&RID=RBJRFKZM013) | ≥99% | Tr |
| *Streptomyces curacoi* | [EF626595.1](http://www.ncbi.nlm.nih.gov/nucleotide/149193340?report=genbank&log$=nucltop&blast_rank=2&RID=WFPZKS9401N) | ≥99% | Cr |
| *Streptomyces niger* | [AB184543.1](http://www.ncbi.nlm.nih.gov/nucleotide/90960364?report=genbank&log$=nucltop&blast_rank=2&RID=VX1C3N9N01N) | ≥99% | Cr |
| *Streptomyces pseudogriseolus* | [HQ850412.1](http://www.ncbi.nlm.nih.gov/nucleotide/325656904?report=genbank&log$=nucltop&blast_rank=1&RID=KXNFMRRY01R) | ≥99% | Cl, Ce |
| *Streptomyces rochei* | [JQ819732.1](http://www.ncbi.nlm.nih.gov/nucleotide/386277653?report=genbank&log$=nucltop&blast_rank=2&RID=VX17820301N) | ≥99% | Cr |
| *Streptomyces tendae* | [HM594286.1](http://www.ncbi.nlm.nih.gov/nucleotide/301790820?report=genbank&log$=nucltop&blast_rank=2&RID=WFNJEV6T012) | ≥99% | Cr |
| *Streptomyces werraensis* | [EU588724.1](http://www.ncbi.nlm.nih.gov/nucleotide/172050870?report=genbank&log$=nucltop&blast_rank=1&RID=KXP62W1801R) | ≥99% | Cl |
| *Weissella viridescens* | [AB680180.1](http://www.ncbi.nlm.nih.gov/nucleotide/359802821?report=genbank&log$=nucltop&blast_rank=2&RID=VX15HE0T01S) | ≥99% | Cr |
|  |  |  |  |
| *Arthrobacter sp.* | FJ766438.1 | ≥99% | Cl |
| *Bacillus sp. (1)* | [AB689744.1](http://www.ncbi.nlm.nih.gov/nucleotide/365733123?report=genbank&log$=nucltop&blast_rank=1&RID=KXNCT57U01R) | ≥99% | Cl |
| *Bacillus sp. (2)* | [AY211142.1](http://www.ncbi.nlm.nih.gov/nucleotide/37785758?report=genbank&log$=nucltop&blast_rank=1&RID=KXNWVCDT01R) | ≥99% | Cl |
| *Burkholderia sp.* | [EF627986.1](http://www.ncbi.nlm.nih.gov/nucleotide/148728555?report=genbank&log$=nucltop&blast_rank=1&RID=RBKW49A4012) | ≥99% | Tr |
| *Cellulosimicrobium sp.* | [AB188222.1](http://www.ncbi.nlm.nih.gov/nucleotide/51491467?report=genbank&log$=nucltop&blast_rank=1&RID=WFNFE8GD016) | ≥99% | Cr |
| *Curtobacterium sp.* | [AY864656.1](http://www.ncbi.nlm.nih.gov/nucleotide/57918786?report=genbank&log$=nucltop&blast_rank=1&RID=WFMPEVW401S) | ≥99% | Cl, Cr |
| *Enterobacter sp.* | [GU726182.1](http://www.ncbi.nlm.nih.gov/nucleotide/312164968?report=genbank&log$=nucltop&blast_rank=1&RID=KACJC3K4016) | ≥99% | Cl, Cr |
| *Erwinia sp.* | [HE646632.1](http://www.ncbi.nlm.nih.gov/nucleotide/363805751?report=genbank&log$=nucltop&blast_rank=1&RID=VX1PGA2F01N) | ≥99% | Cr |
| *Eubacterium sp.* | [AB262669.1](http://www.ncbi.nlm.nih.gov/nucleotide/119371520?report=genbank&log$=nucltop&blast_rank=1&RID=NHYRS6AJ016) | ≥99% | Ce |
| *Klebsiella sp.* | [EU275365.1](http://www.ncbi.nlm.nih.gov/nucleotide/166836210?report=genbank&log$=nucltop&blast_rank=1&RID=VWK5E02E01S) | ≥99% | Cr |
| *Leifsonia sp.* | [JN872512.1](http://www.ncbi.nlm.nih.gov/nucleotide/377551171?report=genbank&log$=nucltop&blast_rank=1&RID=WFMU1UBG016) | ≥99% | Cr |
| *Microbacterium sp.(1)* | [EU362178.1](http://www.ncbi.nlm.nih.gov/nucleotide/169641036?report=genbank&log$=nucltop&blast_rank=1&RID=VWJCMJPX01S) | ≥99% | Cr |
| *Microbacterium sp. (2)* | [FM164624.1](http://www.ncbi.nlm.nih.gov/nucleotide/189406450?report=genbank&log$=nucltop&blast_rank=1&RID=KXN7F8J701S) | ≥99% | Cl |
| *Micromonospora sp.* | [JN120931.1](http://www.ncbi.nlm.nih.gov/nucleotide/347439347?report=genbank&log$=nucltop&blast_rank=1&RID=WFR99PMH012) | ≥99% | Cr |
| *Mycobacterium sp.* | CP002329.1 | ≥99% | Tr |
| *Ochrobactrum sp.* | [AB272074.1](http://www.ncbi.nlm.nih.gov/nucleotide/114213373?report=genbank&log$=nucltop&blast_rank=1&RID=KAUB0ZGT016) | ≥99% | Cl |
| *Paenibacillus sp.(1)* | HE650843.1 | ≥99% | Cl |
| *Paenibacillus sp. (2)* | [HM776635.1](http://www.ncbi.nlm.nih.gov/nucleotide/307564256?report=genbank&log$=nucltop&blast_rank=1&RID=KXN2PEPD01S) | ≥99% | Cl |
| *Ralstonia sp.* | [EU263295.1](http://www.ncbi.nlm.nih.gov/nucleotide/160947035?report=genbank&log$=nucltop&blast_rank=1&RID=RBKTNNFM013) | ≥99% | Tr |
| *Rhodococcus sp.* | [JF734314.1](http://www.ncbi.nlm.nih.gov/nucleotide/333733212?report=genbank&log$=nucltop&blast_rank=1&RID=WFN53SYH013) | ≥99% | Cr |
| *Ruminococcus sp.* | [AB262656.1](http://www.ncbi.nlm.nih.gov/nucleotide/119371515?report=genbank&log$=nucltop&blast_rank=1&RID=NHY26F1K016) | ≥99% | Ce |
| *Streptococcus sp.* | [AB262658.1](http://www.ncbi.nlm.nih.gov/nucleotide/119371517?report=genbank&log$=nucltop&blast_rank=2&RID=KXRM8V6S01S) | ≥99% | Cl, Ce |
|  |  |  |  |
| *Bacillus sp.* | [HM355885.1](http://www.ncbi.nlm.nih.gov/nucleotide/298568602?report=genbank&log$=nucltop&blast_rank=2&RID=KXR4M2AS01S) | 97% | Cl |
| *Chryseobacterium sp.* | [JF722659.1](http://www.ncbi.nlm.nih.gov/nucleotide/332650841?report=genbank&log$=nucltop&blast_rank=1&RID=WFMS9SNW01S) | 98% | Cr |
| *Enterococcus mundtii* | AB602807.1 | 98% | Cl |
| *Lactobacillus ingluviei* | [NR_028810.1](http://www.ncbi.nlm.nih.gov/nucleotide/265678507?report=genbank&log$=nucltop&blast_rank=2&RID=VWJ0YNAR01S) | 98% | Cr |
| *Lactobacillus sp.* | [HM534779.1](http://www.ncbi.nlm.nih.gov/nucleotide/313502597?report=genbank&log$=nucltop&blast_rank=1&RID=VX1YBA8N01S) | 98% | Cr |
| *Oceanobacillus chironomi* | [NR_043700.1](http://www.ncbi.nlm.nih.gov/nucleotide/343203725?report=genbank&log$=nucltop&blast_rank=1&RID=WFR3MS8T012) | 98% | Cr |
| *Paenibacillus glycanilyticus* | [HQ703903.1](http://www.ncbi.nlm.nih.gov/nucleotide/219857131?report=genbank&log$=nucltop&blast_rank=8&RID=49SMG355015) | 98% |  |
| *Paenibacillus pocheonensis* | [AB245386.1](http://www.ncbi.nlm.nih.gov/nucleotide/84453061?report=genbank&log$=nucltop&blast_rank=1&RID=RBK9MAND012) | 98% | Tr |
| *Ruminococcus torques* | [NR_036777.1](http://www.ncbi.nlm.nih.gov/nucleotide/310974914?report=genbank&log$=nucltop&blast_rank=4&RID=NHY9NVN7012) | 97% | Ce |
|  |  |  |  |
| *Actinobaculum massiliense* | [NR_041909.1](http://www.ncbi.nlm.nih.gov/nucleotide/343198486?report=genbank&log$=nucltop&blast_rank=1&RID=KXRHCP6K01S) | 91% | Cl |
| *Actinomyces hyovaginalis* | [NR_026097.1](http://www.ncbi.nlm.nih.gov/nucleotide/219846505?report=genbank&log$=nucltop&blast_rank=1&RID=NHYNVGKC016) | 90% | Ce |
| *Bacillus badius* | [EU221363.1](http://www.ncbi.nlm.nih.gov/nucleotide/161172556?report=genbank&log$=nucltop&blast_rank=4&RID=WFPXNVDC012) | 94% | Cr |
| *Bacteroides plebeius* | [AB200219.1](http://www.ncbi.nlm.nih.gov/nucleotide/67906125?report=genbank&log$=nucltop&blast_rank=2&RID=NHZBEASD012) | 87% | Ce |
| *Clostridium cocleatum* | [AF028350.1](http://www.ncbi.nlm.nih.gov/nucleotide/4205149?report=genbank&log$=nucltop&blast_rank=5&RID=NMB6U3XZ012) | 87% | Cl, Ce |
| *Clostridium sp.* | [AB249652.1](http://www.ncbi.nlm.nih.gov/nucleotide/87239960?report=genbank&log$=nucltop&blast_rank=2&RID=NMB0F0NN013) | 93% | Ce |
| *Corynebacterium falsenii* | AF537594.1 | 96% | Cl |
| *Eubacterium cylindroides* | [AB558487.1](http://www.ncbi.nlm.nih.gov/nucleotide/295315546?report=genbank&log$=nucltop&blast_rank=1&RID=NHYJHGJ1016) | 95% | Ce |
| *Eubacterium oxidoreducens* | [AF202259.1](http://www.ncbi.nlm.nih.gov/nucleotide/6573179?report=genbank&log$=nucltop&blast_rank=2&RID=NHYGGUKB013) | 95% | Ce |
| *Lactobacillus amylovorus* | [CP002609.1](http://www.ncbi.nlm.nih.gov/nucleotide/327182549?report=genbank&log$=nucltop&blast_rank=1&RID=WFTH9FG901S) | 94% | Cr |
| *Lactobacillus gasseri* | [JQ805637.1](http://www.ncbi.nlm.nih.gov/nucleotide/385769697?report=genbank&log$=nucltop&blast_rank=1&RID=VWHFTUFJ01S) | 94% | Ce, Cr, Tr |
| *Lactobacillus intestinalis* | [EF187259.2](http://www.ncbi.nlm.nih.gov/nucleotide/218139555?report=genbank&log$=nucltop&blast_rank=2&RID=WFTF0X2N01S) | 93% | Cr |
| *Leifsonia kribbensis* | NR_044240.1 | 96% | Cl |
| *Negativicoccus succinicivorans* | [FJ715930.1](http://www.ncbi.nlm.nih.gov/nucleotide/224611732?report=genbank&log$=nucltop&blast_rank=1&RID=KXRV33U501S) | 90% | Cl |
| *Ornithinibacillus sp.* | [FJ373040.1](http://www.ncbi.nlm.nih.gov/nucleotide/209552645?report=genbank&log$=nucltop&blast_rank=1&RID=WFMMW0KX013) | 96% | Cr |
| *Paenibacillus chibensis* | [AB681006.1](http://www.ncbi.nlm.nih.gov/nucleotide/359804284?report=genbank&log$=nucltop&blast_rank=6&RID=NHVU4WBS012) | 96% | Ce |
| *Parabacteroides merdae* | [AB238929.1](http://www.ncbi.nlm.nih.gov/nucleotide/89191778?report=genbank&log$=nucltop&blast_rank=1&RID=NMBBBM3K01S) | 93% | Ce |
| *Peptoniphilus sp.* | [FJ577254.2](http://www.ncbi.nlm.nih.gov/nucleotide/237506915?report=genbank&log$=nucltop&blast_rank=1&RID=KXRFV7JW01S) | 91% | Cl |
| *Propionibacterium sp.* | [EU937734.1](http://www.ncbi.nlm.nih.gov/nucleotide/197709098?report=genbank&log$=nucltop&blast_rank=3&RID=KXREH2F501R) | 94% | Cl |
| *Rhizobium sp.* | [FJ405377.1](http://www.ncbi.nlm.nih.gov/nucleotide/212283793?report=genbank&log$=nucltop&blast_rank=1&RID=NHMAS48P016) | 89% | Ce |
| *Streptococcus pluranimalium* | [EF151155.1](http://www.ncbi.nlm.nih.gov/nucleotide/120568240?report=genbank&log$=nucltop&blast_rank=1&RID=VWHXNBZ301N) | 92% | Cr |

*The 16S rRNA Gene Sequences (~500bp) were subjected to homology search against GenBank database by using the BLAST search algorithm as well as MicroSeq ID 2.0 500-bp library. Accession number of the best match and percent of similarity are provided. Tissue type: Ce, cecum, Cr, crop; Cl: cloaca; and Tr: trachea.
